# The G_1_-S transition is promoted by Rb degradation via the E3 ligase UBR5

**DOI:** 10.1126/sciadv.adq6858

**Published:** 2024-10-23

**Authors:** Shuyuan Zhang, Lucas Fuentes Valenzuela, Evgeny Zatulovskiy, Lise Mangiante, Christina Curtis, Jan M. Skotheim

**Affiliations:** ^1^ Department of Biology, Stanford University, Stanford, CA 94305, USA.; ^2^ Department of Biochemistry, University of Cambridge, Cambridge CB2 1GA, UK.; ^3^ Stanford Cancer Institute, Stanford, CA 94305, USA.; ^4^ Chan Zuckerberg Biohub, San Francisco, CA 94158, USA.

## Abstract

Mammalian cells make the decision to divide at the G_1_-S transition in response to diverse signals impinging on the retinoblastoma protein Rb, a cell cycle inhibitor and tumor suppressor. Passage through the G_1_-S transition is initially driven by Rb inactivation via phosphorylation and by Rb’s decreasing concentration in G_1_. While many studies have identified the mechanisms of Rb phosphorylation, the mechanism underlying Rb’s decreasing concentration in G_1_ was unknown. Here, we found that Rb’s concentration decrease in G_1_ requires the E3 ubiquitin ligase UBR5. *UBR5* knockout cells have increased Rb concentration in early G_1_, exhibited a lower G_1_-S transition rate, and are more sensitive to inhibition of cyclin-dependent kinase 4/6 (Cdk4/6). This last observation suggests that UBR5 inhibition can strengthen the efficacy of Cdk4/6 inhibitor–based cancer therapies.

## Teaser

The E3 ligase UBR5 progressively reduces the concentration of the retinoblastoma protein Rb during G_1_ to drive proliferation.

## INTRODUCTION

The decision to divide often takes place in the G_1_ phase of the cell cycle and occurs in response to diverse input signals. Once taken, the decision to initiate DNA replication and divide is difficult to reverse, despite changes to the input signals (*1*, *2*). From a molecular point of view, the commitment point at the G_1_-S transition in response to growth signals corresponds to the hyperphosphorylation and inactivation of the transcriptional inhibitor Rb, the retinoblastoma protein (*1*, *3*, *4*). Hyperphosphorylation of Rb frees the activating E2F transcription factors to drive expression of the cyclins E and A, which can form complexes with the cyclin-dependent kinase Cdk2. Active cyclin E/A–Cdk2 complexes then maintain Rb hyperphosphorylation so that E2F-dependent transcription remains active throughout S phase (*5*). While the molecular basis of the commitment point to cell division is increasingly well understood (*1*, *2*, *6*–*8*), we know much less about how the upstream input signals transmit quantitative information to the decision point.

Multiple input signals regulating the G_1_-S transition operate by inactivating Rb. The best known inputs are the cyclin-Cdk complexes phosphorylating Rb (*5*, *9*–*12*). Upstream growth factors initiate signals that increase the expression of cyclin D (*13*), which primarily forms a complex with the cyclin-dependent kinases Cdk4 and Cdk6 (*14*). cyclin D–Cdk4/6 complexes then initiate the phosphorylation of Rb, possibly through hypo- or monophosphorylation (*15*, *16*). The hypophosphorylation of Rb likely shifts the dissociation constant (*K*_d_) of Rb with E2F, thus promoting the G_1_-S transition. Once Rb is hyperphosphorylated, possibly by the increasing cyclin E–Cdk2 activity and the initial cyclin D–Cdk4/6 activity, it is fully inactivated so that E2F can drive the E2F-dependent S phase transcription program. This Rb phosphorylation pathway is frequently hijacked in cancers to drive cell proliferation (*17*–*21*). For example, cyclin D amplification is frequent in patients with breast cancer and is associated with shorter relapse-free survival (*17*, *22*). Moreover, increasing the cyclin D–Cdk4 activity by transducing cells with a *CDK4* construct is a common approach to immortalizing cells in vitro (*23*, *24*). For many cancer and immortalized cell lines, Rb is immediately hyperphosphorylated after cell birth due to a high Cdk activity likely arising both from mutations promoting proliferation in the rich in vitro cell culture environment (*2*, *25*).

A second input signal that inactivates Rb, which we recently identified, operates through decreasing the concentration of the Rb protein during cell growth in G_1_ (*26*–*28*). Specifically, the total amount of Rb protein stays relatively constant throughout G_1_, while the cell is growing bigger, so that its concentration decreases. In contrast, the concentrations of G_1_-S activators, including cyclin D and E2F, stay constant during early- to mid-G_1_. These differential effects on protein concentration in G_1_ phase drive relative changes in the activities of the cell cycle inhibitor (Rb) and activators (E2F and cyclin D) to favor progression through the G_1_-S transition.

**Fig. 1.**
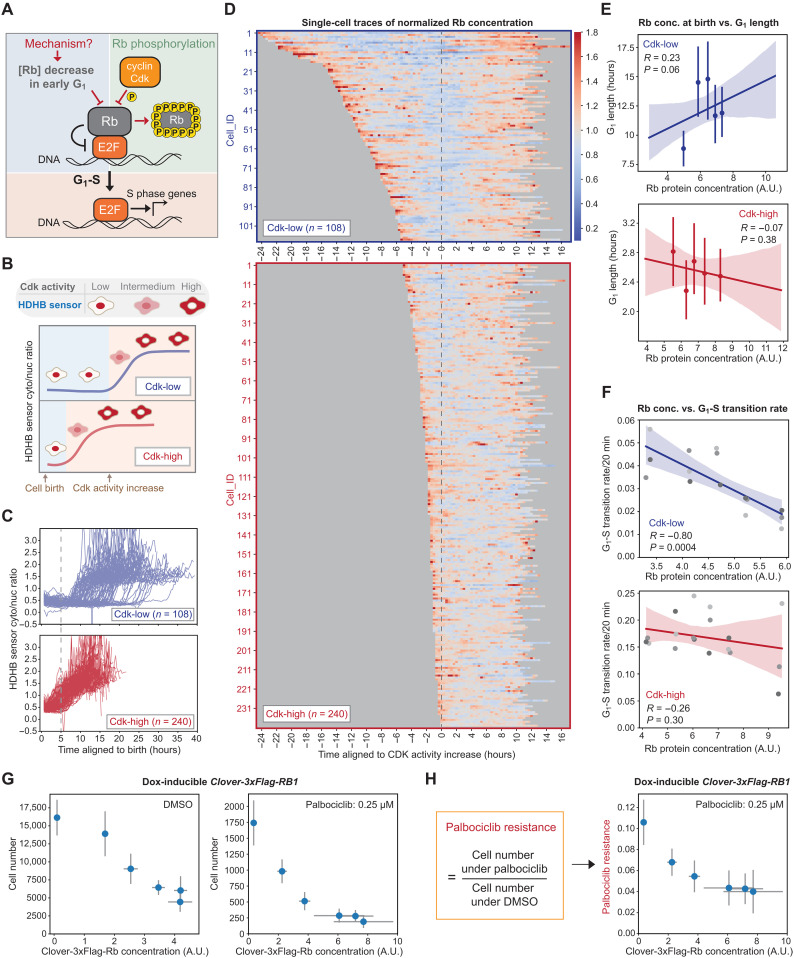
Rb concentration dynamics regulate cell cycle progression in Cdk-low cells. (**A**) Schematic illustration of two parallel mechanisms acting through Rb concentration and Rb phosphorylation to promote the G_1_-S transition. (**B**) Schematic illustration of the Cdk activities in Cdk-low and Cdk-high categories of cells. Cdk activity is measured using the HDHB sensor whose translocation to the cytoplasm from the nucleus is driven by Cdk activity. Cdk activation is defined using the inflection point in the cytoplasm-to-nuclear ratio. (**C**) Fluorescent traces showing the HDHB cytoplasm-to-nucleus intensity ratio from HMEC-hTERT1 cells expressing endogenously tagged *RB1-3xFLAG-Clover-sfGFP* and the HDHB Cdk sensor (*25*). Cdk-high cells are defined as those activating Cdk within 5 hours of their birth, while Cdk-low cells activate Cdk later. (**D**) Individual fluorescent traces from the cells in (C). Top: Cdk-low cells. Bottom: Cdk-high cells. Traces are aligned by the initial translocation of the HDHB Cdk sensor. The trace color represents the normalized Rb protein concentration (normalized to the mean Rb concentration of the cells in the same group). (**E**) The correlations between Rb concentration at birth and G_1_ length in Cdk-low and Cdk-high cells. A.U., arbitrary units. (**F**) The correlations between Rb concentration and the G_1_-S transition rate in Cdk-low and Cdk-high cells. (**G**) Cell number of HMECs treated with 0.25 μM palbociclib for 72 hours. Cells were plated in different doses of Dox to induce exogenous Clover-3xFlag-Rb. Drug treatment started the next day and lasted for 72 hours. Then, cells were fixed, and the cell number in each well was measured. (**H**) Normalized cell number for the cells treated as described in (G). Normalized cell number is the cell number following palbociclib treatment divided by the cell number under DMSO control treatment. *n* = 3 biological replicates. The error bars indicate SD.

Thus, our current model is that two Rb-inactivation input signals cooperate to activate E2F-dependent transcription and initiate the cell cycle (Fig. 1A) (*26*–*28*). Namely, cyclin D–dependent Rb hypophosphorylation shifts the *K*_d_ of Rb with E2F so that the decreasing Rb concentration can drop below *K*_d_ to release active E2F. Following the G_1_-S transition, Rb’s concentration increases during S-G_2_-M to reset for the next cell cycle. Unlike the Cdk-dependent pathway, the dilution of Rb protein does not depend on any increased Cdk activity and can serve as a parallel mechanism to inactivate Rb and promote the G_1_-S transition. Although several molecular mechanisms underlying cyclin D synthesis and Rb phosphorylation have been elucidated (*15*, *29*), the molecular mechanism underlying Rb’s concentration decrease as cells progress through G_1_ is unknown.

Here, we sought to determine the molecular mechanisms underlying Rb’s concentration decrease through G_1_ (Fig. 1A). We found that Rb is consistently synthesized throughout the cell cycle, but its degradation is cell cycle dependent. Specifically, Rb is targeted for degradation in G_1_ by the E3 ligase UBR5 and stabilized by hyperphosphorylation at the G_1_-S transition. A mathematical model shows that this mechanism is sufficient to explain the observed concentration dynamics through the cell cycle. Disruption of this Rb degradation mechanism via UBR5 deletion decreases the G_1_-S transition rate and sensitizes cells to chemical inhibition of Cdk4/6 activity. This last observation suggests the efficacy of Cdk4/6 inhibitor–based therapies could be improved through targeting UBR5. Indeed, *UBR5* is frequently amplified in patients with breast cancer and this amplification is associated with worse prognosis.

## RESULTS

### Rb concentration dynamics regulate cell cycle progression in cells born with low Cdk activity

While the decreasing Rb concentration in G_1_ drove cell cycle progression in some cells (*26*, *27*), many cell lines appeared to not respond to changes in Rb dosage in terms of their proliferation rate (*16*, *30*). We therefore sought to further test the effect of Rb concentration on cell cycle progression and identify the reason behind this apparent discrepancy. To do this, we performed live-cell imaging on *HMEC-hTERT1* cells (telomerase-immortalized human mammary epithelial cells, abbreviated as HMECs) expressing endogenously tagged *RB1-3xFLAG-Clover-sfGFP* and the HDHB (human DNA helicase B) Cdk activity sensor (*25*). The nuclear-to-cytoplasm translocation of this fluorescent sensor marks the transition point in mid/late-G_1_ when Cdk activity abruptly increases (Fig. 1B) (*1*, *25*). On the basis of how quickly the Cdk activity rises after birth, we classified the cells into two categories: Cdk-high cells and Cdk-low cells based on whether Cdk activity had risen 5 hours after birth (Fig. 1, B and C, and fig. S1C). This classification has been used in other studies that applied the same Cdk sensor on MCF10A cells (*25*). When we aligned the cell traces to the inflection point of the HDHB sensor that marks Cdk activity increase, we found that the concentration of Rb continuously decreases during early- to mid-G_1_ phase in the Cdk-low cells and then increases through the remainder of the cell cycle (Fig. 1D and fig. S1, A, B, and D to G). However, Cdk-high cells do not exhibit decreasing Rb concentration dynamics in G_1_ (Fig. 1D and fig. S1, A, B, and D to G). Thus, an Rb concentration decrease in G_1_ could only regulate the cell cycle in Cdk-low cells, but not in Cdk-high cells, where the concentration decrease does not take place and Rb is likely rapidly inactivated.

To test whether the decrease in Rb concentration promotes cell cycle entry in Cdk-low cells, we examined the relationship between Rb concentration with the G_1_ duration and the G_1_-S transition rate. We found that the Rb concentration at birth is positively correlated with G_1_ duration (Fig. 1E), and that Rb concentration is anticorrelated with the G_1_-S transition rate (Fig. 1F). That these correlations are significant in Cdk-low cells but not in Cdk-high cells is consistent with Rb concentration regulating the G_1_-S transition only when Cdk activity is low. To further test this by exogenously controlling the concentration of Rb, we used HMECs containing a doxycycline (Dox)–inducible allele of *RB1*. Increasing Rb concentration not only suppresses cell proliferation but also sensitizes cells to treatment by Cdk4/6 inhibitors (Fig. 1, G and H, and fig. S2). Together, these experiments are consistent with our previous work and a recent study reporting that decreasing Rb concentration in G_1_ plays a crucial role in driving cells into the cell cycle in the absence of Cdk4/6 activity and facilitates the adaptation to chemical Cdk4/6 inhibitors (*31*).

Our findings demonstrate that Rb concentration more significantly affects cells with initially low Cdk activity. This makes sense because cells born with high Cdk activity likely quickly inactivate Rb via phosphorylation. This observation also explains why, in many experiments, overexpression of Rb does not have a big impact on cell proliferation (*16*, *32*). This is likely because most cultured cell lines, especially cancer cell lines, are in the Cdk-high category (*25*, *32*). Even in the noncancer cell lines, such as the telomerase-immortalized HMECs we used here, most cells are born with high Cdk activity. However, this is not true in cells growing in vivo. For example, the epidermal stem cells in mouse skin tissue have a much longer G_1_ duration than in vitro cultured cell lines and are not born with high Cdk activity (*28*, *33*). Therefore, the Rb concentration is likely to have a bigger impact on cell cycle progression in vivo.

### Rb concentration dynamics are driven by cell cycle–dependent protein degradation

**Fig. 2.**
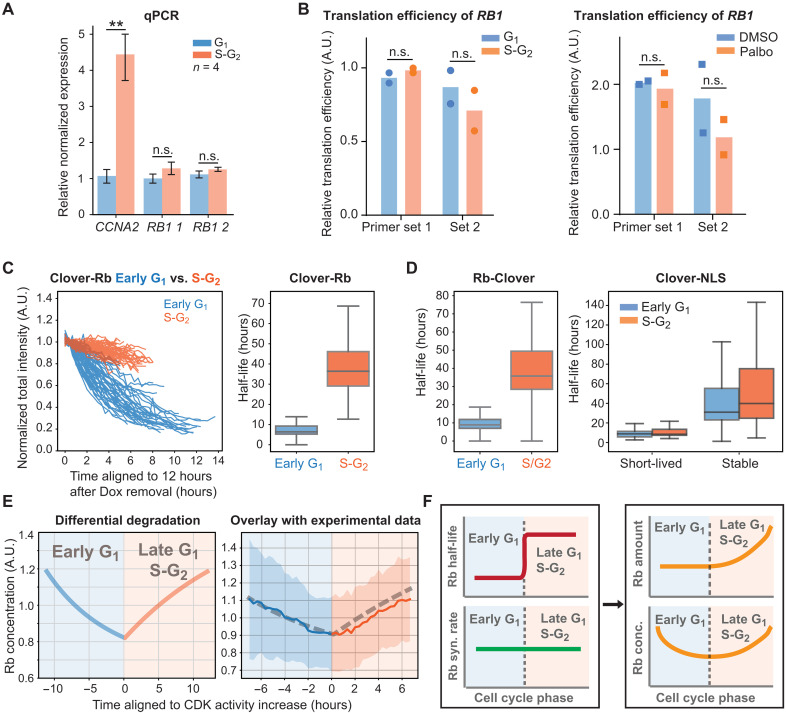
Rb concentration dynamics are driven by cell cycle–dependent protein degradation. (**A**) qPCR (*n* = 4) measurements of the *RB1* mRNA concentration in G_1_ and S-G_2_ HMECs sorted using a FUCCI cell cycle reporter. **P < 0.01. n.s., not significant. (**B**) Translation efficiency of *RB1* was determined in HMECs in G_1_ and S-G_2_ that had been sorted using a FUCCI marker (left) and in HMECs treated with DMSO or palbociclib (1 μM) (right). Translation efficiency was measured using an RIP assay by pulling down eIF4E. Translation efficiency is calculated by dividing the eIF4E-bound fraction of *RB1* mRNA to the eIF4E-bound fraction of *GAPDH* and *Actin* mRNAs. Bars denote mean values, and dots denote each replicate experiment. (**C**) Left: The degradation traces of Clover-3xFlag-Rb protein from individual cells following Dox withdrawal. The traces were classified into early G_1_ phase or S-G_2_ phase based on a FUCCI cell cycle marker and cell cycle phase duration. Right: Distribution of half-lives estimated from exponential fits. Box plot indicates 5th, 25th, median, 75th, and 95th percentiles. (**D**) Same half-life measurement as in (C), but using a C-terminally tagged Rb-3xFlag-Clover (left), a short-lived Clover-NLS, and a stable Clover-NLS (right). (**E**) Left: Rb concentration dynamics calculated assuming that its degradation rate decreases by 80% at the G_1_-S transition as measured by live imaging [see (C)] and its synthesis rate does not change. Right: Overlay of the model based on regulated Rb degradation with the experimental data from fig. S1D (Cdk-low cells). Rb concentration is normalized to the mean. (**F**) Model schematic: Differential degradation of Rb in G_1_ and S-G_2_ phases of the cell cycle drive its concentration dynamics.

To identify the mechanism regulating Rb concentration dynamics through the cell cycle, we examined both the synthesis and degradation of Rb protein in different cell cycle phases. We measured the mRNA concentration of *RB1* in different cell cycle phases using flow cytometry to sort HMECs expressing FUCCI (*34*) cell cycle reporters into G_1_ and S-G_2_ populations. We then performed quantitative polymerase chain reaction (qPCR) and mRNA sequencing to measure *RB1* mRNA. The results showed that the *RB1* mRNA concentration did not significantly increase in S-G_2_ phase (Fig. 2A and fig. S3A). We found a similar result when calculating *RB1* mRNA concentrations in different cell cycle phases using a published MERFISH dataset (*35*) (fig. S3B). This indicates that Rb concentration dynamics are controlled by posttranscriptional mechanisms. To further investigate the synthesis dynamics of Rb protein, we measured the translation efficiency of *RB1* mRNA by performing a RIP (RNA binding protein immunoprecipitation) assay against the translation initiation factor eIF4E (eukaryotic translation initiation factor 4E) (*36*). The relative translation efficiency is calculated by dividing the bound fraction of *RB1* with the bound fraction of housekeeping genes (*Actin* and *GAPDH*). Using this method, we found that the relative translation efficiency of *RB1* was similar in sorted G_1_ and S-G_2_ cells and in asynchronously dividing and G_1_-arrested cells (palbociclib treatment) (Fig. 2B and fig. S3C). Together, our data indicate that Rb synthesis is not primarily responsible for its cell cycle dynamics.

Having found that Rb’s cell cycle dynamics were not primarily due to transcription or translation mechanisms, we next sought to test whether protein degradation was responsible. To do this, we used a Dox-inducible system in which cells conditionally express Clover-3xFlag–tagged Rb (*TRE-Clover-3xFlag-Rb*) or Clover-NLS (nuclear localization signal) (*TRE-Clover-NLS*) upon Dox treatment (1 μg/ml). After 36 hours of Dox treatment, we withdrew Dox and monitored the decrease in the Clover fluorescence signal using live-cell imaging (fig. S4A). Since the cells also express a FUCCI cell cycle marker, we can separately assess protein degradation taking place in G_1_ and S-G_2_ phases of the cell cycle (fig. S4A). By fitting the degradation traces using a simple exponential decay function, we estimated the half-life of Clover-3xFlag-Rb protein in different cell cycle phases for each cell. Rb half-life in early G_1_ (median, 6.4 hours; 75% range, 5.3 to 9.4 hours) is significantly shorter than it is in S-G_2_ (median, 37.3 hours; 75% range, 29.2 to 45.4 hours) (Fig. 2C). The Rb tag location does not affect its half-life since a C-terminally tagged Rb protein (Rb-3xFlag-Clover) behaved similarly to the N-terminally tagged version (Fig. 2D). The changing protein stability in G_1_ compared to S-G_2_ phases was specific to Rb as the short-lived Clover-NLS protein and stable Clover-NLS protein expressed with the same Dox-inducible system both had half-lives that did not change through the cell cycle (Fig. 2D and fig. S4, B and C). Thus, these results suggest Rb’s cell cycle dynamics are due to its degradation in G_1_ and stabilization at the G_1_-S transition.

Having established that the regulation of Rb stability is most likely responsible for its cell cycle dynamics, we sought to test whether this differential degradation of Rb is sufficient to give rise to the observed dynamics. To do this, we generated a mathematical model where only the half-life of the Rb protein changed through the cell cycle, while the synthesis rate remained constant (see Materials and Methods). This simple model revealed that regulated degradation was sufficient to generate the cell cycle–dependent Rb concentration dynamics we observed, while the modest up-regulation in the synthesis rate was insufficient (Fig. 2E and fig. S4D). Note that the “Rb protein dilution” phenomenon that we previously observed in (*26*), in which the total Rb protein amount is kept at a constant level during early G_1_ while the cell size is growing bigger, is a result of the changing balance between Rb synthesis and Rb being more actively degraded during early G_1_. From the model, we also examined the dynamics of total Rb protein amount, and we found that, with the experimental parameters for synthesis and degradation rates, the Rb protein amount is relatively constant in early G_1_ phase (fig. S4E). This shows that the observed dilution in G_1_ can be explained by Rb’s degradation rate being similar to its synthesis rate in G_1_, while cell size increases. Together, our data and analysis indicate that the cell cycle–dependent regulation of Rb stability is primarily responsible for its cell cycle dynamics (Fig. 2F).

### Rb is stabilized via phosphorylation by Cdk

**Fig. 3.**
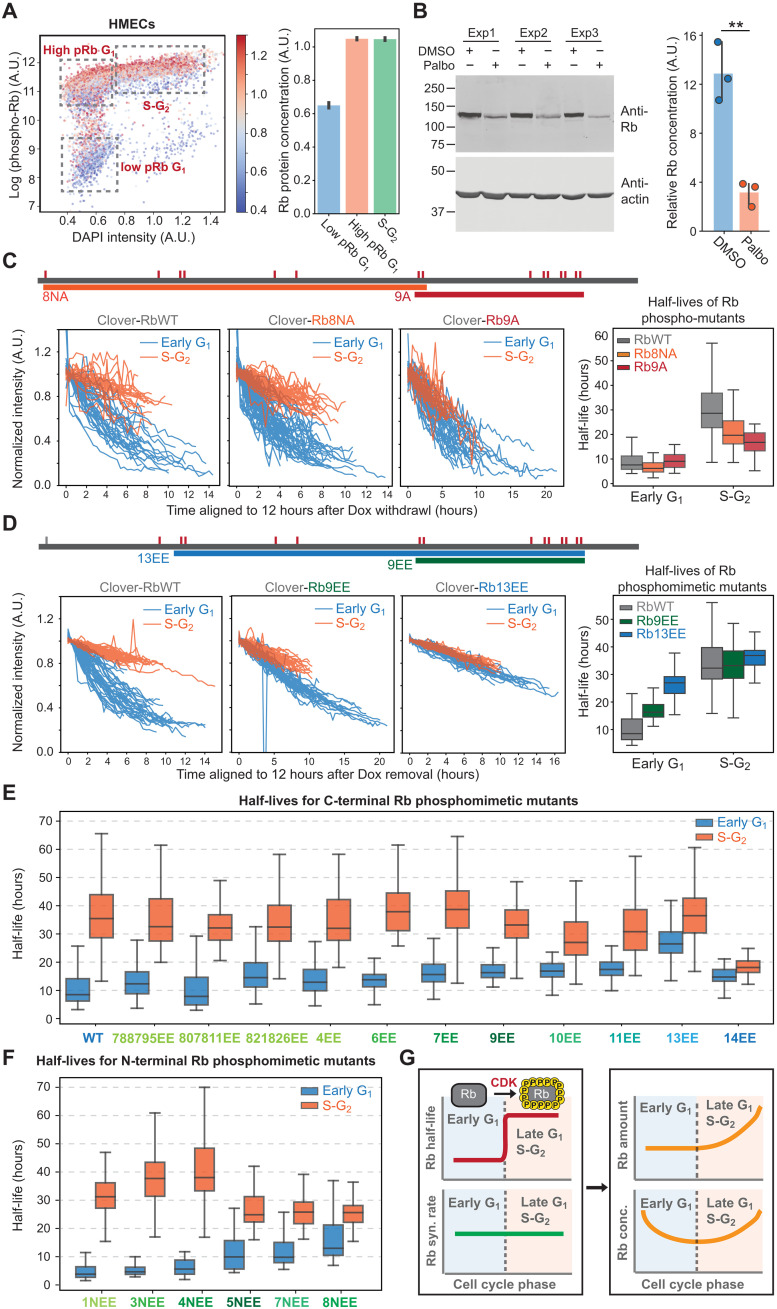
Rb protein is stabilized via phosphorylation by cyclin-Cdk. (**A**) Rb concentration in different pRb populations. HMECs were stained with pRb (S807/811) and Rb antibodies. Left: pRb intensity is plotted against DNA content [4′,6-diamidino-2-phenylindole (DAPI) intensity]. The color indicates Rb concentration. Right: Quantification of Rb concentrations in different pRb populations gated using the indicated boxes in the left panel. Bar plots indicate the mean and 95% confidence interval. (**B**) Immunoblot of Rb after DMSO or palbociclib (1 μM) treatment for 24 hours. The quantification of relative Rb concentration (normalized to actin intensity) is shown on the right. **P < 0.01. (**C**) Top: Schematic of Rb phospho-site mutants. Small red lines indicate the location of Cdk phosphorylation sites. Bottom: Degradation traces for Clover-3xFlag-RbWT, Clover-3xFlag-Rb8NA, and Clover-3xFlag-Rb9A, as well as the corresponding distributions of half-lives. (**D**) Top: Schematic of the Rb phosphomimetic mutants. Bottom: Degradation traces for Clover-3xFlag-RbWT, Clover-3xFlag-Rb9EE, and Clover-3xFlag-Rb13EE, as well as the corresponding distributions of half-lives. (**E** and **F**) Half-life distributions for all the Rb phosphomimetic mutants. (**G**) Model schematic: Rb is stabilized in late G_1_ and S-G_2_ phases by Cdk phosphorylation.

Having found that Rb is stabilized at the G_1_-S transition, we next sought to identify the molecular mechanism. One of the most prominent molecular changes occurring at the G_1_-S transition is the phosphorylation of Rb by cyclin-Cdk complexes. We therefore sought to examine how Rb phosphorylation affected its half-life. To do this, we first stained asynchronous HMECs with phospho-Rb (S807/811) (pRb) and total Rb antibodies. We then calculated the Rb concentration in the low pRb G_1_ population, the high pRb G_1_ population, and the S-G_2_ population. The Rb concentration is lower in early G_1_ when it is not hyperphosphorylated and then begins to recover in late G_1_, when Rb is hyperphosphorylated (Fig. 3A). Similar results were obtained when G_1_ was partitioned into early and late phases using a recently published live-cell Cdk activity sensor (KTR sensor) based on the C-terminal part of Rb (886 to 928 amino acids of Rb) (fig. S5A) (*37*). These immunofluorescence data support the model where the Rb’s concentration decrease in G_1_ phase is reversed upon its hyperphosphorylation. Consistently, when cells are arrested in G_1_ by treating them with the Cdk4/6 inhibitor palbociclib (1 μM) for 24 hours, the Rb protein concentration drops by about 75% (Fig. 3B) although the mRNA concentration is only reduced by about 15% (fig. S5B). This is consistent with published results showing significant Rb protein drops when cells are exposed to Cdk4/6 inhibitors (*38*). Furthermore, in cells expressing a Dox-inducible Clover-3xFlag-Rb protein, palbociclib treatment led to a significant decrease in the concentration of this ectopically expressed protein but not the corresponding mRNA (fig. S5C). Together, these data suggest that the phosphorylation of Rb by Cdk mediates its stabilization. To test this model, we used the HDHB Cdk sensor to categorize cells into Cdk-low and Cdk-high populations before measuring the half-lives of Clover-Rb and Clover-NLS (fig. S5, D and E). As anticipated, we found that Rb was degraded much more rapidly in the Cdk-low population.

To further investigate how Rb phosphorylation on different Cdk phosphorylation sites affects its half-life, we used the Dox-inducible system to express a series of Rb variants in which the Cdk phosphorylation sites were either substituted with nonphosphorylatable alanines or with phosphomimetic double glutamic acid residues (EE) (fig. S6A) (*39*). For both mutant series, we extended the number of mutant sites from either the N or C terminus so that different mutants covered different parts of the protein (fig. S6A). If Cdk phosphorylation stabilizes Rb, then the phospho-mutants (S/T to A) should exhibit a reduced half-life in S-G_2_, and the phosphomimetic mutants (S/TP to EE) should exhibit an increased half-life in early G_1_. Our results are consistent with this hypothesis (Fig. 3, C and D and fig. S6, B and C). Note that the C-terminal alanine mutants also had a more severe cell cycle arrest phenotype (fig. S7A). This is likely because the alanine mutants do not allow the phosphorylation of C-terminal residues to disrupt Rb’s interaction with E2F-DP (Dimerization Partner) (*11*, *12*, *29*, *40*). On the other hand, the phosphomimetic mutants did not demonstrate significant cell cycle defects (fig. S7B), likely because these Rb mutants are partially or entirely unable to bind and inhibit E2F. In addition, the introduction of phosphomimetic mutations resulted in a smaller Rb’s concentration decrease in cells arrested in G_1_ using palbociclib (fig. S7, C and D).

Our mutational analysis did not reveal any particular phosphorylation sites that predominantly regulated Rb’s half-life (Fig. 3, E and F, and figs. S6C and S7, C and D). Instead, the degree of Rb stabilization, namely the ratio between early G_1_ and S-G_2_ half-lives, correlated with the total number of phosphomimetic sites. This shows that many different phosphorylation sites contribute to Rb stability. We note that Rb14EE exhibited a reduced half-life in both early G_1_ and S-G_2_ phases, which is likely due to the additional SP230EE mutation destabilizing the protein via another mechanism (Fig. 3E and fig. S7D). However, the difference between early G_1_ and S-G_2_ half-lives in Rb14EE is the smallest (fig. S6C). Collectively, these results support the hypothesis that Rb is stabilized by its hyperphosphorylation in late G_1_ by Cdk complexes (Fig. 3G).

### The degradation of un- or hypophosphorylated Rb is mediated by the E3 ubiquitin ligase UBR5

**Fig. 4.**
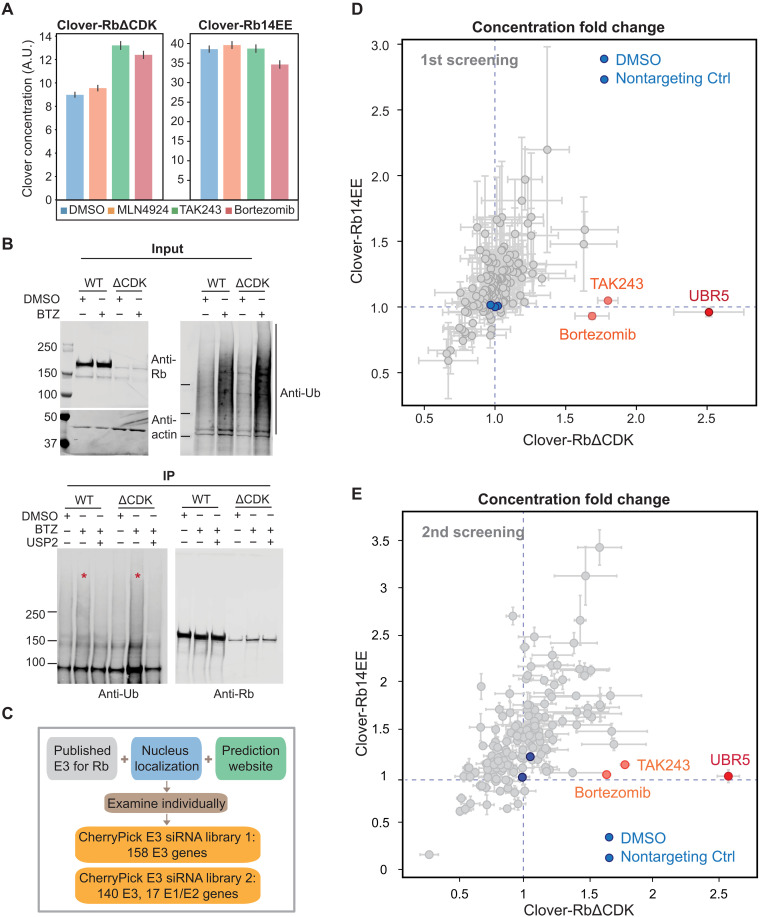
siRNA screens identified UBR5 as the E3 ligase targeting unphosphorylated Rb for degradation. (**A**) Concentrations of Clover-3xFlag-RbΔCDK or Clover-3xFlag-Rb14EE after drug treatments. Cells expressing Clover-3xFlag-RbΔCDK and Clover-3xFlag-Rb14EE were induced with Dox (1 μg/ml) for 48 hours. Then, cells were treated with the indicated drugs for 5 hours, fixed, and imaged. The concentrations of Clover-3xFlag-Rb variants were calculated by dividing total Clover intensity by nuclear area^3/2^. (**B**) Unphosphorylated Rb is degraded via ubiquitination (Ub). HMECs expressing Clover-3xFlag-RbΔCDK or RbWT [induced by Dox (1 μg/ml) for 48 hours] were treated with bortezomib (BTZ; 1 μM) or DMSO for 5 hours before collection. Clover-3xFlag-RbΔCDK or RbWT proteins were immunoprecipitated (IP) using anti-Flag magnetic beads. After purification, the bortezomib-treated samples were split in half, and one-half underwent a deubiquitination assay using USP2 (ubiquitin specific peptidase 2). The samples were then detected for Rb and ubiquitin using immunoblotting. (**C**) Schematics showing siRNA library components. (**D** and **E**) Results of the first and second siRNA screens. The concentration fold changes of Clover-3xFlag-RbΔCDK and Clover-3xFlag-Rb14EE are plotted. The fold change is calculated by dividing the Clover concentration of the treatment well by the concentration of the nontreated well. *n* = 4 biological replicates for the first screen and *n* = 3 biological replicates for the second screen.

After establishing that Rb is stabilized by phosphorylation at the G_1_-S transition, we next sought to identify the underlying molecular mechanism. To do this, we first tested whether Rb is degraded through the ubiquitin-proteasome system by treating cells with three commonly used inhibitors targeting different components of this degradation system: Bortezomib inhibits the proteasome; TAK243 inhibits the ubiquitin activating enzyme (E1); and MLN4924 inhibits the NEDD8-activating enzyme that activates the Cullin (CUL)–RING E3 ubiquitin ligases (*41*, *42*). We treated asynchronously growing HMECs with these inhibitors for 5 hours and then immunostained the cells using antibodies for pRb (S807/811) and total Rb. TAK243 and bortezomib treatments increased the Rb concentration in the low pRb G_1_ populations to a level similar to that in the high pRb G_1_ population, but MLN4924 did not (fig. S8, A and B). RPE-1 cells (telomerase-immortalized retinal pigment epithelium cells) behaved similarly to HMECs in that only TAK243 and bortezomib treatments increased the Rb concentration in the low pRb G_1_ population (fig. S8C). To further confirm that the phosphorylation status determines Rb degradation through the ubiquitin-proteasome system, we similarly treated cells that were induced to express unphosphorylated Rb that lacks all its Cdk phosphorylation sites (Clover-3xFlag-RbΔCDK) or phosphomimetic Rb (Clover-3xFlag-Rb14EE). The concentration of RbΔCDK is elevated by TAK243 and bortezomib, but not MLN4924, and the concentration of Rb14EE does not increase following treatment by any of the three inhibitors (Fig. 4A). We also confirmed the enhanced ubiquitination of RbΔCDK by pulling down Clover-3xFlag-RbΔCDK and blotting for ubiquitin. RbΔCDK was more ubiquitinated than wild-type (WT) Rb, which is mostly in the hyperphosphorylated form (Fig. 4B). We also performed the anti-ubiquitin pull-down and blotted for Rb. Although the signals are weak, there is more RbΔCDK in the ubiquitinated proteins than RbWT or Rb13EE (fig. S8D). Together, these data suggest that unphosphorylated Rb is degraded in G_1_ through the ubiquitin-proteasome system, but not by the CUL-RING E3 ligases.

There have been several previous studies of Rb degradation mechanisms that identified some E3 ligases (*38*, *43*–*50*). For example, MDM2 (mouse double minute 2) may mediate Rb degradation via its central acidic domain (*43*, *44*, *48*). The human papilloma virus E7 protein can bind Rb and induce its degradation (*47*), which is mediated by protease cleavage at K810 (*45*). More recently, Cdk4/6 inhibition was found to promote Rb degradation through the F-box protein βTrCP1-mediated ubiquitination (*38*). To test whether these E3 ligases were responsible for the observed cell cycle dynamics of Rb, we examined the effect of knocking them down on the concentration of unphosphorylated Rb (Clover-3xFlag-RbΔCDK) and phosphomimetic Rb (Clover-3xFlag-Rb14EE). If an E3 were responsible for Rb’s cell cycle dynamics, then we would expect to see an increase in the concentration of Clover-3xFlag-RbΔCDK but not of Clover-3xFlag-Rb14EE. None of the knockdowns exhibited this predicted phenotype (fig. S9A). Even through some of the knockdowns affected the overall Rb concentration, this effect was not specific for unphosphorylated Rb and, therefore, could not explain Rb’s cell cycle dynamics. Similarly, we performed the same set of knockdowns in cells arrested in G_1_ using palbociclib and did not find any specific increase in the concentrations of un- or hypophosphorylated Rb (fig. S9, B to G). This implies that there must be some additional E3 ligase responsible for the phosphorylation-dependent degradation of Rb.

To identify the E3 ligases mediating the degradation of unphosphorylated Rb, we set up a small interfering RNA (siRNA) screen. We used a customized siRNA library that included the previously published E3 ligases for Rb, some nuclear localized E3s (according to UniProt), and some additional genes predicted to be E3 ligases for Rb (http://ubibrowser.bio-it.cn/ubibrowser_v3/) (Fig. 4C). HMECs inducibly expressing Clover-3xFlag-RbΔCDK or Clover-3xFlag-Rb14EE were transfected with the siRNA library. Forty-eight hours later, cells were fixed and imaged. The concentration of Clover-3xFlag-Rb variants was measured in each treatment, and its fold change over nontransfected cells was calculated. As positive controls, we included the ubiquitin-proteasome system inhibitors TAK243 and bortezomib. As expected, TAK243 and bortezomib only increased the concentration of RbΔCDK but not Rb14EE (Fig. 4D and fig. S10, A and B). From this screen, we only identified UBR5 as specifically targeting unphosphorylated Rb for degradation (Fig. 4D and fig. S10, A and B). UBR5 is a verified E3 ubiquitin ligase belonging to the HECT (homologous to the E6AP carboxyl terminus) family known to play roles in transcription and the DNA damage response (*51*–*54*). However, Rb has never been reported to be a substrate of UBR5. To confirm that UBR5 is the main E3 ligase targeting unphosphorylated Rb, we first performed another siRNA screen with a different siRNA library containing UBR5 and 17 other E3 genes from the first library as well as the rest of the nuclear localized E3 genes not included in the first screen. We also included several E1 and E2 genes (Fig. 4C). This second siRNA screen also only identified UBR5 (Fig. 4E and fig. S10, B to D).

**Fig. 5.**
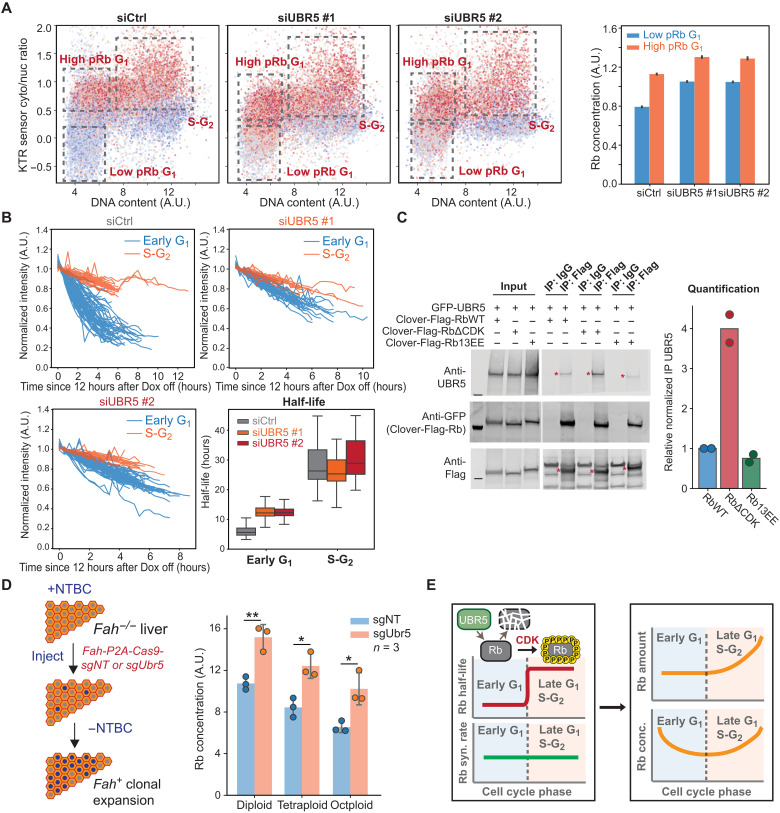
Validation of UBR5 as the E3 ligase mediating the degradation of unphosphorylated Rb. (**A**) Microscopy analysis of HMECs expressing the Rb (886 to 928) KTR sensor (*37*), which reflects Cdk activity. Cells were treated with control siRNA or *UBR5* siRNAs for 48 hours. The cytoplasm-to-nucleus intensity ratio of the KTR sensor is plotted against DNA content (DAPI staining). The dot color indicates the Rb concentration. Quantification of the Rb concentration for different pRb levels is shown on the right. Bars indicate mean and the 95% confidence interval. (**B**) Degradation traces and the calculated half-lives of Clover-3xFlag-Rb after *UBR5* knockdown. Box plot indicates 5th, 25th, median, 75th, and 95th percentiles. (**C**) Unphosphorylated Rb interacts with the E3 ligase UBR5. Human embryonic kidney (HEK) 293 cells were transfected with a Clover-3xFlag-RbWT, RbΔCDK, or Rb13EE construct and a green fluorescent protein (GFP)–UBR5 construct (Addgene, #52050). Twenty-four hours after transfection, cells were induced by Dox. After 24 hours of induction, cells were lysed, and Flag antibodies were used to pull down Clover-3xFlag-Rb mutants. The samples were then detected for UBR5 and Rb using immunoblotting. For quantification, the coimmunoprecipitated UBR5 was normalized to the immunoprecipitated Rb mutant protein amount and the input UBR5 protein amount. (**D**) Left: Schematic of the *Fah^−/−^* mouse liver model. Right: Rb concentration in the low-pRb population of the primary hepatocytes isolated from mice receiving *Fah-P2A-Cas9-sgNT* or *Fah-P2A-Cas9-sgUbr5* transposons. The error bars indicate the SD of the mean. (**E**) Model schematic: Unphosphorylated Rb is targeted for degradation by the E3 ligase UBR5 in early G_1_.

We then validated UBR5 as a hit using another two independent siRNAs against UBR5. Knockdown of UBR5 in HMECs led to the accumulation of un/hypophosphorylated Rb after palbociclib treatment, as measured by both immunoblotting and immunostaining (Fig. 5A and fig. S11A). We also measured the half-life of Rb following UBR5 knockdown using live-cell imaging and found that Rb was degraded about twice as slowly in early G_1_, but there was no change in its stability in S-G_2_ (Fig. 5B). Moreover, we examined the effect of knocking down UBR5 on HMECs expressing endogenously tagged Rb (*RB1-3xFLAG-Clover-sfGFP*) (*26*). Following *UBR5* knockdown, the concentration of Rb does not decrease in early G_1_ but is instead kept relatively constant (fig. S11B). To further confirm that the degradation of unphosphorylated Rb by UBR5 is not cell line or cell type specific, we also examined epithelial RPE-1 cells, HLF (primary human lung fibroblast), and T98G (glioblastoma-derived fibroblast-like) cells. All of them showed that UBR5 knockdown increased concentrations of un- and hypophosphorylated Rb in palbociclib-treated cells (fig. S11, C to E).

After establishing that Rb degradation depends on UBR5, we sought to test for a direct interaction between these two proteins. To do this, we performed immunoprecipitation assays with UBR5 and unphosphorylated Rb, WT Rb, and phosphomimetic Rb. We found that the unphosphorylated Rb, which lacks all Cdk sites, can pull down significantly more UBR5 than WT Rb. Moreover, the phosphomimetic Rb pulled down the smallest amount of UBR5 (Fig. 5C). This result showing a direct phosphorylation-dependent interaction is consistent with our hypothesis that UBR5 targets unphosphorylated Rb for degradation and Rb phosphorylation protects Rb from UBR5-dependent degradation.

To test whether UBR5 mediated Rb degradation in vivo, we examined its effect in the mouse liver using the *Fah^−/−^* system (*55*, *56*). In the *Fah^−/−^* system, deletion of the *Fah* gene causes toxin accumulation in hepatocytes that will lead to hepatocyte death. Toxin accumulation can be prevented by treating mice with NTBC [2-(2-nitro-4-trifluoromethylbenzoyl)-1,3-cyclohexanedione] (*56*). When NTBC is withdrawn, cells expressing exogenous *Fah*, introduced by injecting *Fah^+^* transposons, will clonally expand to repopulate the injured liver (Fig. 5D) (*56*). Other genetic elements, such as Cas9 and guide RNA, can be added to the *Fah* transposon so that they are cointegrated into some hepatocytes genomes. To knock out *Ubr5* in some hepatocytes, we modified an *Fah* transposon plasmid (*57*) and delivered *Fah-P2A-Cas9-sgUbr5* or *Fah-P2A-Cas9-sgNT* (nontargeting) transposons and the SB100 transposase into *Fah^−/−^* mice via hydrodynamic transfection. Eight weeks after injection, when the liver was almost fully repopulated with *Fah^+^* cells, we isolated the hepatocytes, plated them, and performed immunostaining or immunoblotting (fig. S11F). Consistent with the results from human cell lines, knocking out *Ubr5* increased Rb concentrations in mouse hepatocytes where Rb was not hyperphosphorylated (low pRb) (Fig. 5D and fig. S11, G and H). The results from this *Fah^−/−^* in vivo model further support our conclusion that the E3 ligase UBR5 targets unphosphorylated Rb for degradation in G_1_ (Fig. 5E).

Our results here give insight into why previous studies reported other E3s targeting Rb. First, any E3 whose knockdown results in a cell cycle phenotype would be predicted to have an effect on Rb concentration. Second, after finding that Rb dynamics were driven by the degradation of un- or hypophosphorylated Rb in G_1_, we sought to find E3s that specifically targeted the unphosphorylated RbΔCDK protein, but not the phosphomimetic Rb14EE protein. We did find a significant increase in both RbΔCDK and Rb14EE when the E3 MDM2 was knocked down and possibly very modest effects when other reported E3s were knocked down (figs. S9A and S10A). This suggests that these other E3s might operate in different cell types or contexts but are not generally responsible for Rb’s cell cycle dynamics.

Last, we note that depletion of UBR5 did not fully restore the Rb half-life in G_1_ to a similar level as that in S-G_2_ (Fig. 5D and fig. S13, A to D), suggesting that other E3 ligases might be involved to degrade the unphosphorylated Rb. Further supporting the hypothesis that additional E3s are involved in Rb degradation, the treatment of cells with the proteasome inhibitor bortezomib further stabilized Rb in both G_1_ and S-G_2_ phases (fig. S13, E and F). Thus, UBR5 is unlikely to be the only E3 ligase targeting un- and hypophosphorylated Rb for degradation. However, UBR5 is likely to be the E3 responsible for the most unphosphorylated or hypophosphorylated Rb protein degradation.

### UBR5 and Cdk4/6 are distinct inputs promoting the G_1_-S transition

**Fig. 6.**
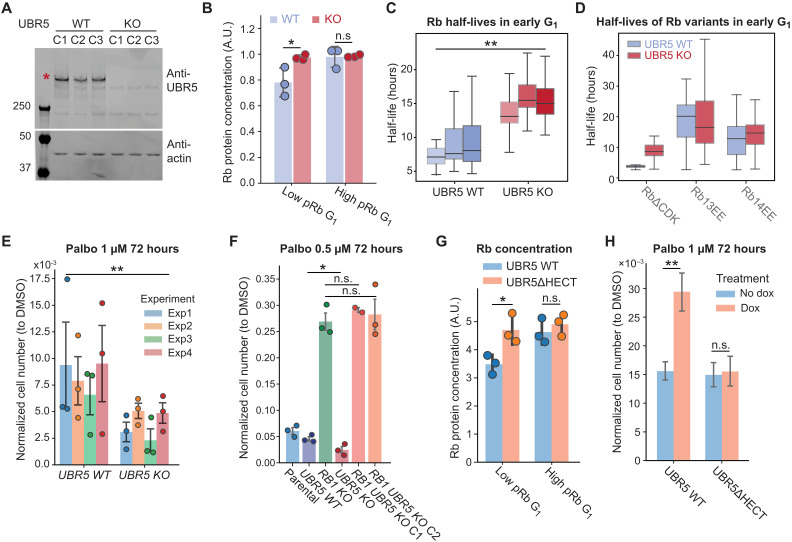
UBR5 deletion stabilizes Rb and sensitizes cells to CDK4/6 inhibition. (**A**) Immunoblot verification of *UBR5 KO* clonal cell lines. (**B**) Quantification of Rb concentration in different pRb populations of *UBR5 WT* or *KO* clonal cell lines expressing the Rb (886 to 928) KTR sensor (*37*). Circles denote results from individual clones, and the bars denote the SD. (**C**) The half-life distributions of Clover-3xFlag-RbWT protein in early G_1_ phase from six clonal cell lines (three *UBR5 WT* cells and three *UBR5 KO* cells). (**D**) The half-life distributions of Clover-3xFlag-RbΔCDK, Rb13EE, and Rb14EE in early G_1_ phase in *UBR5 WT* and *UBR5 KO* cells. (**E**) Normalized cell number of *UBR5 WT* and *KO* clonal cell lines after palbociclib (1 μM) treatment for 72 hours. Normalized cell number is the cell number following a palbociclib treatment divided by the cell number following a DMSO control treatment. *n* = 4 biological replicates. Error bars indicate the SD. (**F**) Normalized cell number for *UBR5 WT*, *RB1 KO*, and *RB1 UBR5* double *KO* clonal cell lines following palbociclib (0.5 μM) treatment for 72 hours. *n* = 3 biological replicates. (**G**) Quantification of Rb concentration in different pRb populations of *UBR5 KO* cells with either *UBR5 WT* or *UBR5ΔHECT* added back by Dox induction. Circles denote results from individual clones, and the bars denote the SD. (**H**) Normalized cell number of *UBR5 KO* cells with *UBR5 WT* or *UBR5ΔHECT* added back following palbociclib (1 μM) treatment for 72 hours. The results show the average from three *UBR5 KO* clonal cell lines with different UBR5 variants added back. *n* = 3 biological replicates. Error bars indicate the SD. *P < 0.05 and **P < 0.01.

It is becoming increasingly clear that there are two distinct signal inputs driving the G_1_-S transition that both operate through Rb. First, cyclin D–Cdk4/6 complexes phosphorylate and inhibit Rb, and, second, Rb degradation drives down the concentration of Rb in G_1_ phase. One prediction from this parallel input model is that cells lacking Rb degradation should be more sensitive to inhibition of Rb phosphorylation. We can now test this prediction because we identified UBR5 as the E3 ligase mediating the degradation of unphosphorylated or hypophosphorylated Rb in G_1_. To do this, we first generated clonal cell lines lacking *UBR5* from HMECs using CRISPR-Cas9. We randomly picked three *UBR5 WT* clones and three *UBR5 KO* (knockout) clones for analysis (Fig. 6A). *UBR5 KO* cells exhibited higher Rb concentrations in low pRb G_1_ cells (Fig. 6B and fig. S12A). Moreover, *UBR5 KO* cells also exhibited both higher endogenous Rb concentrations and higher exogenous Clover-3xFlag-Rb concentrations following palbociclib treatment to arrest cells in G_1_ (figs. S12, B and C, and S14, A and B). *UBR5 KO* cells also exhibited increases in Rb half-life in early G_1_ (Fig. 6C and fig. S13, A and B),and exhibited increases in the half-life of unphosphorylated Rb∆CDK, but not phosphomimetic Rb14EE, as measured by our live-cell imaging assay (Fig. 6D and fig. S13, C and D). These knockout lines therefore exhibited all the same effects we observed in our earlier knockdown experiments shown in Fig. 5.

Having generated *UBR5 KO* cells, we can now test the parallel input model prediction that cells lacking the Rb degradation mechanism are more sensitive to the inhibition of Rb phosphorylation. To do this, we treated *UBR5 WT* and *UBR5 KO* cells with the Cdk4/6 inhibitor palbociclib for 72 hours and then measured cell proliferation by counting cell numbers. Since different clonal cell lines had different proliferation rates to begin with (fig. S15A), we normalized the cell numbers of palbociclib-treated cells to the cell numbers in the dimethyl sulfoxide (DMSO) control treatment for each clonal cell line. Moreover, this normalization also accounts for the slower growth rates of *UBR5 KO* cells (fig. S15A) that are likely due to the dysregulation of other UBR5 substrates. As predicted by the parallel input model, *UBR5 KO* cells are more sensitive to palbociclib treatment than *UBR5 WT* cells (Fig. 6E and fig. S15A). To further determine the proliferation status of *UBR5 WT* and *KO* cells, we also stained the cells with pRb antibodies following prolonged DMSO or palbociclib treatment up to 6 days. As expected, a significantly higher proportion of *UBR5 WT* cells were progressing through the cell cycle (as indicated by cells having hyperphosphorylated Rb) compared to *UBR5 KO* cells, again supporting the parallel input model (fig. S15, B to E).

Since UBR5 has other substrates that may also affect cell cycle progression, we wanted to examine whether *UBR5 KO* cells’ increased sensitivity to palbociclib treatment was due to the stabilization of Rb. To test this, we knocked out *RB1* in *UBR5 KO* cells using CRISPR-Cas9 (fig. S16A) and tested their sensitivity to palbociclib treatment. Knocking out *RB1* in *UBR5 KO* cells completely rescued the increased palbociclib sensitivity exhibited by *UBR5 KO* cells (Fig. 6F and fig. S16B), suggesting that the effect of UBR5 on the G_1_-S transition is primarily through the stabilization of Rb. Last, to make sure that the effect of UBR5 on cell cycle progression was due to its E3 ligase activity, we added back either WT UBR5 or an inactive mutant UBR5 to the *UBR5 KO* cells using the Dox-inducible system (fig. S17A). The mutant UBR5 has a C2768A mutation in the HECT domain (abbreviated as UBR5∆HECT), which kills its catalytic activity (*52*). Consistent with the role of UBR5 degrading unphosphorylated or hypophosphorylated Rb, the expression of UBR5 WT was able to reduce Rb concentration in the low pRb G_1_ population in *UBR5 KO* cells, but expressing UBR5∆HECT did not (Fig. 6G and fig. S17, B and C). We treated cells expressing UBR5 WT or UBR5∆HECT (induced by Dox) with DMSO or palbociclib for 72 hours and found that adding back UBR5 WT decreased the cells’ palbociclib sensitivity compared to the no Dox control, whereas adding back the UBR5∆HECT did not (Fig. 6H and fig. S18, A and B). This indicates that the E3 ligase activity of UBR5 is essential for Rb degradation.

**Fig. 7.**
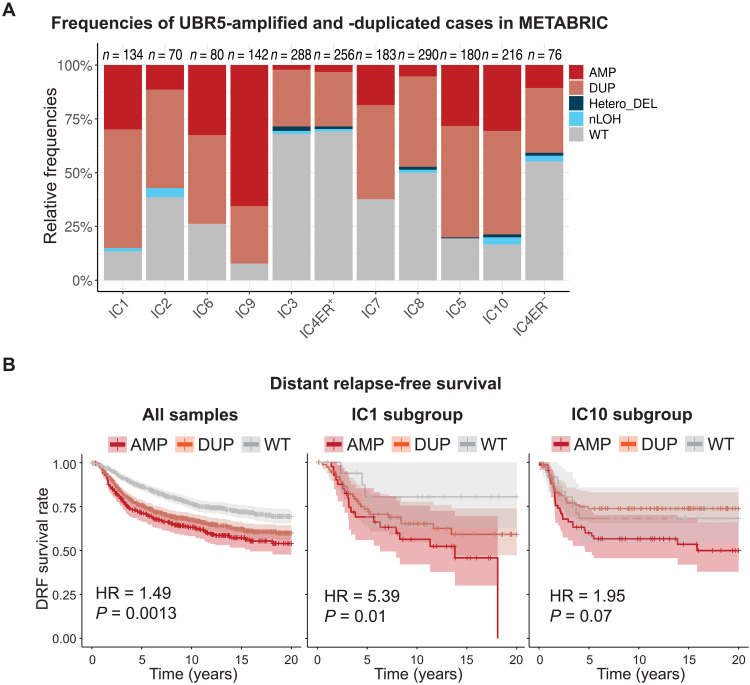
*UBR5* amplification is frequent in patients with breast cancer and is associated with worse prognosis. (**A**) Frequencies of *UBR5* amplification in the METABRIC dataset across the different breast cancer subtypes. The number of tumors within each group is indicated on the top of each bar. AMP, amplification with a total copy number ≥ 6; DUP, duplication with a total copy number between 3 and 5; Hetero_DEL, heterozygous deletion; nLOH, neutral loss of heterozygosity. (**B**) Kaplan-Meier curves of DRF survival for the *UBR5*-amplified, *UBR5*-duplicated, and WT cases across all samples (left), in the IC1 subgroup (middle), and in the IC10 subgroup (right). The squares correspond to estimated hazard ratios, and the segments correspond to their 95% confidence intervals. HR, hazard ratio. *P* value tests the difference between the WT and the AMP curves corrected for the other clinical covariates (*59*).

Since deleting UBR5 sensitizes cells to treatment by Cdk4/6 inhibitors that are currently used to target breast cancers, we next explored whether UBR5 itself could be a potential therapeutic target. To do this, we examined the *UBR5* copy number level in patients with breast cancer and how it relates to the patient’s survival. Since there is a lot of variability between patients depending on the molecular basis of their disease, we categorized patients for analysis using the Integrative Cluster subtype (IC) framework that partitions groups of patients based on multiple types of genomic data (*58*, *59*). IC subtypes predict patient outcomes beyond the historical clinical stratification based on hormone receptors status: estrogen receptor–positive (ER^+^), human epidermal growth factor receptor-2–positive (HER2^+^), and triple-negative subgroups (*58*, *59*). IC classification stratifies ER^+^ tumors into ER^+^ typical risk (IC3, IC4ER^+^, IC7, and IC8) and ER^+^ high risk of relapse (IC1, IC2, IC6, and IC9) categories and stratifies triple-negative breast cancer (TNBC) into the genomically stable and unstable subtypes IC4ER^−^ and IC10, respectively (*58*, *59*). Among the 1894 patient samples from the Molecular Taxonomy of Breast Cancer International Consortium (METABRIC), the *UBR5* gene was amplified in most patients. When stratified by the prognostic subtypes, *UBR5* gene copy amplification was observed in all subgroups but to various degrees (Fig. 7A and fig. S18C). *UBR5* amplification was particularly dominant in subgroups associated with a worse prognosis (ER^+^ high risk, HER2^+^, and TNBC tumors). We then assessed how *UBR5* copy number alteration affected patient survival. By analyzing the distant relapse-free (DRF) survival of these patients, we found that *UBR5* amplification is associated with worse DRF survival (Fig. 7B), especially in IC1 (ER^+^ high risk) and IC10 (TNBC and basal-like tumors) subtypes (Fig. 7B). These results suggest that breast cancer cells might increase *UBR5* expression through copy number amplification to facilitate proliferation.

## DISCUSSION

**Fig. 8.**
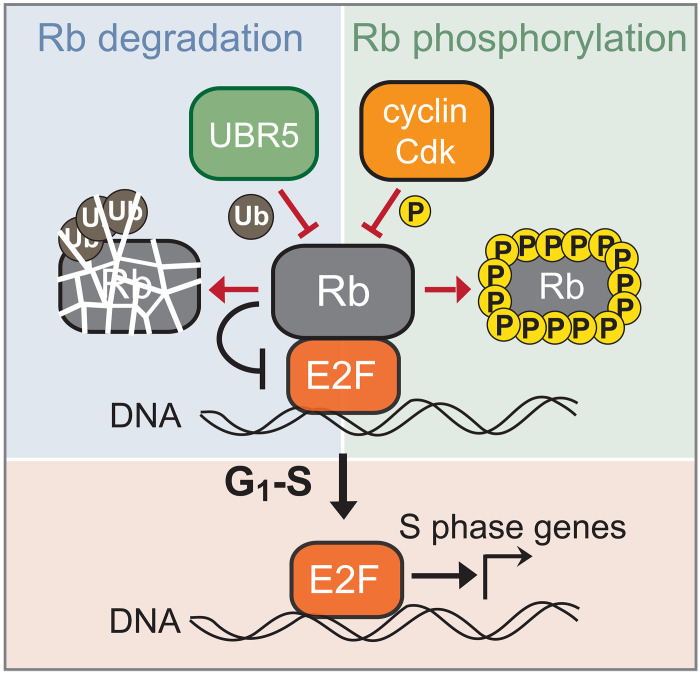
Model schematic of parallel inputs regulating Rb activity and thereby the G_1_-S transition. Rb is inhibited by phosphorylation and targeted for degradation by UBR5.

Two distinct input signals drive the G_1_-S transition by reducing the activity and concentration of the key cell cycle inhibitor Rb (Fig. 8). Here, we report that the decreasing concentration of Rb in early G_1_ is driven by its degradation. Rb degradation is promoted by the E3 ubiquitin ligase UBR5 targeting un- and hypophosphorylated Rb. Rb accumulation then takes place following its hyperphosphorylation in late G_1_. This mechanism could explain why some cells can still progress into S phase and activate Cdk2 even in the absence of cyclin D–dependent kinase activity (*7*, *60*, *61*). Namely, these cells rely on UBR5-mediated Rb degradation. In addition to providing such a robust entry to the cell division cycle in a particular context, the existence of multiple input signals regulating the G_1_-S transition might be due to the different proliferative requirements of diverse cell types (*62*).

While the two signaling inputs impinging on Rb activity use distinct mechanisms, degradation, and phosphorylation, their activities become interconnected once cells begin the G_1_-S transition. Namely, the degradation of Rb eventually drives an increase in E2F activity and, thereby, transcription of cyclin A and cyclin E. These downstream cyclins can then form a complex with Cdk2 to drive Rb hyperphosphorylation and stabilization. Consequently, Rb degradation drives Cdk activity, which, in turn, inhibits Rb degradation as cells progress into S phase. Nevertheless, while these activities become interconnected at the restriction point and the G_1_-S transition, before this point, it is likely that they operate independently so that UBR5 and cyclin D–Cdk4/6 activities can be separately modulated to control the timing of the G_1_-S transition in a variety of cellular contexts.

Our finding that *UBR5* promotes the degradation of Rb in G_1_ phase raises several questions. First, does UBR5 directly ubiquitinate Rb? Can we isolate the role of UBR5 targeting Rb from the role of UBR5 targeting its other substrates? What other E3 ligases play a role in Rb degradation? If UBR5 directly ubiquitinates Rb, then it should be possible to recreate Rb ubiquitination in vitro and to identify the molecular docking site on Rb. If such a docking site can be identified and mutated, then one could disentangle the role of UBR5-mediated Rb degradation from UBR5’s role in targeting other substrates such as *MYC* (*52*–*54*). UBR5 likely engages its substrates as a dimer or tetramer, which can target distinct degron linear motifs as indicated by recent cryo–electron microscopy structures (*53*, *54*, *63*–*65*). Two recent studies proposed that UBR5 targets its substrates on chromatin (*53*, *54*). This possible preference of UBR5 for chromatin-bound targets might explain the results of our mutational analysis of Rb. Namely, the more tightly an Rb variant is predicted to bind the E2F transcription factor, the more rapidly it is degraded. The Rb variants with increasing numbers of phosphomimetic sites (Fig. 3E and fig. S6, A and C) have decreasing binding affinity to E2F (and chromatin) and exhibit increasing stability in G_1_. In support of such a model, the stabilization of Rb at the G_1_-S transition is coincident with its hyperphosphorylation and dissociation from the chromatin-bound E2F transcription factors (*66*). Last, we note that UBR5 is unlikely to be the only E3 ligase that mediates the degradation of Rb because depletion of UBR5 did not fully stabilize Rb (Fig. 5B and fig. S13).

UBR5 appears frequently mutated in cancer and may become a target for therapies similarly to other G_1_-S regulators (*67*). Cdk4/6 inhibitors in combination with endocrine therapy are used to treat advanced ER^+^/HER2^−^ breast cancers (*68*–*70*). However, this application is frequently limited by the intrinsic and acquired therapeutic resistance observed in patients (*71*, *72*). One possible way to improve upon current therapies targeting the Rb phosphorylation pathway is to also target the degradation of Rb through UBR5. Since deleting UBR5 sensitizes cells to treatment by Cdk4/6 inhibitors, it is possible that current Cdk4/6 inhibitor–based treatments for breast cancer can be improved by developing novel therapeutics targeting Rb degradation through UBR5, which is frequently amplified in these cancers (Fig. 6I).

The inability of current therapies to inhibit cell division most likely reflects our incomplete knowledge of the signaling pathways involved. Here, we report a previously unknown mechanism regulating Rb degradation that cells can use in combination with cyclin D–Cdk4/6 activity. These two signals can be used together to differentially regulate proliferation to satisfy the requirements of the myriad cell types of a multicellular organism. Thus, a better understanding of the molecular mechanisms underlying the G_1_-S transition will give us insight into both development and disease.

## MATERIALS AND METHODS

### Mice

All work was performed in accordance with the guidelines of the Institutional Animal Care and Use Committee at Stanford University (protocol number: APLAC 33191). The *Fah^−/−^* mice were shared by M. Grompe’s group at Oregon Health & Science University. At 6 weeks old, the *Fah^−/−^* mice were subjected to hydrodynamical transfection through their tail vein. We injected one plasmid containing a transposon that carries *Fah*, *Cas9*, and a guide RNA and a second plasmid containing the transposase SB100. The plasmid backbone was provided by H. Zhu’s laboratory at UT Southwestern Medical Center. We modified the guide RNA sequence on the transposons to either a negative control guide RNA sequence or a guide RNA targeting the *Ubr5* gene. Eight weeks after the injection, the mice were euthanized to isolate primary hepatocytes. The study has been approved.

### Cell culture conditions and cell lines

All cells were cultured at 37°C with 5% CO_2_. Nontransformed hTERT1-immortalized HMECs were obtained from S. Elledge’s laboratory at Harvard Medical School (*73*) and cultured in MEGM mammary epithelial cell growth medium (Lonza, CC-3150). In microscopy experiments, we used the same medium but without phenol red to reduce background fluorescence (Lonza CC-3153 phenol red–free basal medium supplemented with growth factors and other components from the Lonza CC4136 kit). T98G cells were purchased from American Type Culture Collection, recently isolated primary fetal HLFs were purchased from Cell Applications, and hTERT1-immortalized retinal pigment epithelium (RPE-1) cells were obtained from the Cyert laboratory at Stanford. All these cell lines were grown in Dulbecco’s modified Eagle’s medium with l-glutamine, glucose (4.5 g/liter), and sodium pyruvate (Corning), supplemented with 10% fetal bovine serum (Corning) and 1% penicillin/streptomycin.

### Fluorescent reporter cell lines

The S-G_2_ component of the FUCCI cell cycle reporter—mCherry-Geminin—was cloned into the CSII-EF-MCS lentiviral vector backbone under a constitutive EF1α promoter (*1*). The CSII vector, the lentiviral packaging vector dr8.74, and the envelope vector VSV-G (Vesicular stomatitis virus G) were transfected into human embryonic kidney (HEK) 293T cells by polyethylenimine (PEI) (1 mg/ml; Sigma-Aldrich). Forty-eight hours later, the lentivirus-containing medium was collected and used to infect HMECs. Two to 3 days after infection, positive cells were sorted by fluorescence-activated cell sorting (FACS) and expanded. The endogenously tagged *RB1-3xFLAG-Clover-sfGFP* HMEC cell line was created by Zatulovskiy *et al.* (*26*).

### Inducible expression of WT and mutant Rb in cell lines

To inducibly express WT Rb and Rb mutants in cells, we used the Dox-inducible Rb cassette published in (*29*) and performed site-directed mutagenesis (New England Biolabs, E0554S) to generate Rb mutant plasmids. All the plasmids contained the WT *RB1* gene or *RB1* mutants fused with fluorescent Clover and 3xFLAG affinity tag sequences, a zeocin resistance gene, and a Tet-On 3G transactivator gene driven by the EF1α promoter. The HMEC cell lines stably expressing Dox-inducible Rb variants were generated by transfecting cells with 1 μg of Dox-inducible plasmid and 1 μg of PiggyBac transposase plasmid using the FuGene HD reagent (Promega, E2311). Zeocin (300 μg/ml) selection began 2 days after transfection and lasted for at least 2 weeks until all the cells became resistant.

### *UBR5* knockout cell lines and *RB1* knockout cell lines

*UBR5* knockout HMECs and *RB1* knockout HMECs were generated using the CRISPR knockout kit v2 from Synthego following the manufacturer’s protocol. Briefly, the ribonucleoprotein (RNP) complexes (9:1 single guide RNA–to–Cas9 ratio) were assembled in Nucleofector solution plus supplement (Lonza Amaxa HMEC Nucleofector Kit) to a total volume of 100 μl. The RNP was incubated at room temperature for 10 min. A total of 0.5 million HMECs were resuspended in 100 μl of RNP solution and transferred to a cuvette. This was followed by electroporation using the Lonza Nucleofector 2b device (program Y-001). Forty-eight hours later, the knockout efficiency was examined by immunoblot. In both cases, we obtained about 40 to 50% knockout efficiency. The resulting cell populations were then single cell sorted to make clonal cell lines. After single-cell expansion, the grown out clones were validated for *RB1* or *UBR5* knockout, and WT clones and knockout clones were kept for further analysis. The *UBR5 RB1* double knockout cell lines were generated by knocking out the *RB1* gene in the *UBR5* knockout clones.

### Primary hepatocyte isolation and two-dimensional culture

Primary hepatocytes were isolated by two-step collagenase perfusion (*74*) using liver perfusion medium (Thermo Fisher Scientific, 17701038), liver digest medium (Thermo Fisher Scientific, 17703034), and hepatocyte wash medium (Thermo Fisher Scientific, 17704024). The protocol for two-dimensional culture of primary hepatocytes was shared by Y. Jin from R. Nusse’s laboratory at Stanford University (*75*). Briefly, primary hepatocytes from *Fah^−/−^* mice were isolated by two-step collagenase perfusion. After isolation, cells were washed three times with hepatocyte wash medium (Thermo Fisher Scientific, 17704024). Cells were then plated in a six-well plate precoated with collagen I (50 μg/ml) at a density of 200,000 cells per well. The culture medium contained 3 μM CHIR99021 (Peprotech), epidermal growth factor (25 ng/ml; Peprotech), hepatocyte growth factor (50 ng/ml; Peprotech), and tumor necrosis factor–α (100 ng/ml; Peprotech) in basal medium. The basal medium contained William’s E medium (Gibco), 1% GlutaMAX (Gibco), 1% nonessential amino acids (Gibco), 1% penicillin/streptomycin (Gibco), 0.2% normocin (Invitrogen), 2% B27 (Gibco), 1% N2 supplement (Gibco), 2% fetal bovine serum (Corning), 10 mM nicotinamide (Sigma-Aldrich), 1.25 mM *N*-acetylcysteine (Sigma-Aldrich), 10 μM Y27632 (Peprotech), and 1 μM A83-01 (Tocris). The culture medium was refreshed every other day. Cells were passaged via trypsinization using TrypLE (Thermo Fisher Scientific). The first passage cells were used for immunostaining or Western blot analysis.

### Live-cell imaging and analysis

The cells for imaging were seeded on 35-mm glass-bottom dishes (MatTek) 1 day before imaging. Then, the cells were transferred to a Zeiss Axio Observer Z1 microscope equipped with an incubation chamber and imaged for 48 hours at 37°C and 5% CO_2_. Bright-field and fluorescence images were collected at multiple positions every 20 min using an automated stage controlled by the Micro-Manager software. We used a Zyla 5.5 scientific complementary metal-oxide semiconductor camera and an A-plan 10×/0.25 numerical aperture Ph1 objective. For half-life measurements, cells were plated in Dox containing medium (1 μg/ml) and induced for 36 hours. After this induction, the Dox-containing medium was removed, the cells were washed once with fresh Dox-free medium, and then, once the fresh medium lacking Dox was added, the cells were transferred to the microscope for imaging. For most of the Rb mutants, the half-life analysis started 12 hours after Dox removal to eliminate the potential effect of protein synthesis from residual mRNA. For the fast degraded Rb mutants, e.g., RbΔCDK, the half-life analysis started 3 hours after Dox removal to ensure the signal-to-noise ratio is good enough for accurate quantification. The cell cycle stage was classified using an mCherry-Geminin FUCCI sensor. The early G_1_ phase traces were taken as those having no mCherry-Geminin expression that lasted longer than 7 hours. The S-G_2_ phase is defined by mCherry-Geminin FUCCI marker expression. For cells expressing the HDHB Cdk sensor (*25*), the transition from low Cdk activity to high Cdk activity was taken as the inflection point of the cytoplasm-to-nuclear fluorescence ratio (*25*). The volume of cell nucleus was used as a proxy of total cell volume because nuclear volume is known to scale in proportion to cell volume and the nucleus can be segmented and measured much more accurately than the irregular-shaped cell (*76*).

### siRNA transfection

For siRNA screening, the library was purchased from Horizon Discovery. We constructed two customized libraries against the target genes listed in Table S1. We used ON-TARGETplus siRNA (Horizon Discovery), with four different siRNA sequences targeting the same gene pooled together. siRNA transfection was performed using Lipofectamine RNAiMAX (Invitrogen) following the manufacturer’s protocol. Briefly, for reverse transfection, 7500 HMECs with Clover-3xFlag-Rb variant cassettes (15,000 cells if the variant is RbΔCDK because the cells will be arrested in G_1_) suspended in 100 μl of Dox-containing medium (1 μg/ml) were added per well into a 96-well plate containing a mixture of pooled siRNAs (1.5 pmol per well) and Lipofectamine RNAiMAX (0.2 μl per well in 20 μl of Opti-MEM). Cells were subsequently grown for 48 hours at 37°C before fixation. For MLN4924, TAK243, and bortezomib treatments, the drugs were added 5 hours before fixation. Cells were fixed with 4% paraformaldehyde for 20 min at room temperature, permeabilized with 0.5% Triton X-100 (Sigma-Aldrich) for 15 min at room temperature, and then incubated with 500 nM 4′,6-diamidino-2-phenylindole (DAPI) for 30 min at room temperature. After washing with PBS, cells were imaged using the ImageXpress Micro Confocal at the High-Throughput Screening Knowledge Center of Stanford.

For regular siRNA knockdown, we used the Silencer-Select pre-designed siRNA (Ambion, Thermo Fisher Scientific). For most genes, we purchased two different siRNAs. Lipofectamine RNAiMAX (Invitrogen) was used for siRNA transfection. For 24-well plates, cells were plated 1 day before such that they were ~40% confluent at the time of transfection. For each well, 6 pmol of siRNA in 50 μl of Opti-MEM was mixed with 1 μl of RNAiMAX in 50 μl of Opti-MEM. After 10 to 20 min of incubation at room temperature, the mixture was added to the cells. Forty-eight hours later, the cells were lysed for Western blot or qPCR analysis.

### Palbociclib sensitivity assay

Cells were plated in 96-well plates (2000 per well) 1 day before drug treatment. For cells with inducible Rb cassettes, cells were plated in different concentrations of Dox (0, 20, 50, 150, 500, and 1000 ng/ml) to induce different concentrations of Clover-3xFlag-Rb. Palbociclib or DMSO was added the next day, and the medium was refreshed every day. After 3 days of drug treatment, cells were fixed with 4% paraformaldehyde for 20 min at room temperature, permeabilized with 0.5% Triton X-100 (Sigma-Aldrich) for 15 min at room temperature, and then incubated with 500 nM DAPI for 30 min at room temperature before imaging. After washing with PBS, cells were imaged using the ImageXpress Micro Confocal at the High-Throughput Screening Knowledge Center of Stanford. Cell nuclei were segmented and counted to indicate cell number. Four technical replicates were prepared for each condition (four wells for the same genotype and same treatment), and the average was used for the final cell count.

### Immunofluorescence staining

Cells were seeded on a 35-mm glass-bottom dish (MatTek) or a 6/24-well glass-bottom plate (Cellvis) 1 day before immunofluorescence staining. For this staining, cells were fixed with 4% paraformaldehyde for 20 min at room temperature, permeabilized with 0.5% Triton X-100 (Sigma-Aldrich) for 15 min at room temperature, and then blocked with 3% bovine serum albumin (BSA) in PBS. Then, the cells were incubated with primary antibodies overnight at 4°C. After three washes with PBS, the cells were incubated with Alexa Fluor secondary antibodies (Invitrogen, A32728) at 1:1000 for 1 hour at room temperature. After three washes with PBS, cells were incubated with 500 nM DAPI for 30 min at room temperature before imaging. The primary antibodies used for immunofluorescence were anti-Rb (Santa Cruz Biotechnology, sc-74570; 1:100), anti-pRb (S807/811) (Cell Signaling Technology, 8516; 1:400). The cells were imaged using a Zeiss Axio Observer Z1 microscope with an A-plan 10×/0.25 numerical aperture objective. For the 24-well plates, cells were imaged using the ImageXpress Micro Confocal from the High-Throughput Screening Knowledge Center at Stanford.

### Flow cytometry and cell sorting

For flow cytometry analysis, cells were grown on six-well plates to ~70% confluence and harvested following trypsinization. The cells were then fixed with 4% formaldehyde for 10 min at 37°C and permeabilized with 90% ice-cold methanol for 30 min on ice. Fixed and permeabilized cells were washed once with PBS, blocked with 3% BSA in PBS for 30 min at 37°C, and then stained with primary antibodies for 2 hours at 37°C. The cells were then washed twice with a wash buffer (1% BSA in PBS + 0.05% Tween 20), stained with the fluorophore-conjugated secondary antibodies (Life Technologies) at 1:1000 dilution for 1 hour at 37°C, and then washed twice again. After this treatment, the cells were resuspended in PBS containing 3 μM DAPI for DNA staining, incubated for 30 min at room temperature, and then analyzed on an Attune NxT Flow Cytometer (Thermo Fisher Scientific). For live-cell flow cytometry analysis, cells were harvested from dishes by trypsinization, stained with 20 μM Hoechst 33342 DNA dye in PBS for 30 min at 37°C, and then analyzed on an Attune NxT Flow Cytometer (Thermo Fisher Scientific). For live-cell sorting, the cells were harvested from dishes following trypsinization, resuspended in fresh medium, stained with Hoechst 33342 (if sorting by cell cycle phase), and then sorted on a BD FACSAria flow cytometer. DNA content and the mCherry-Geminin fluorescent reporter were used to determine cell cycle phase, and the side scatter area parameter was used as a readout for cell size. During sorting, cell samples were kept at 4°C, and the sorted cells were collected for further RNA isolation and reverse transcription (RT) qPCR analysis or for immunoblotting. For single-clone derivation, cells were sorted into 48-well plates containing growth medium using the single-cell sorting mode.

### RNA immunoprecipitation assay

We performed RNA immunoprecipitation following published protocols (*36*). Briefly, cells were lysed in an ice cold polysome lysis buffer with 3 million to 5 million cells per 1 ml of lysis buffer. The lysate was spun down at 16,000*g* for 15 min at 4°C, and the supernatant was transferred to a new tube. Fifty microliters of equilibrated protein A/G-Agarose beads (Pierce, Thermo Fisher Scientific) were added to the supernatant for preclearing at 4°C for 1 hour. One hundred microliters of the lysate was saved as the input sample, and the rest was incubated with 5 μg of eIF4E antibody (Santa Cruz Biotechnology, sc-271480) or mouse immunoglobulin G (IgG) (Santa Cruz Biotechnology, sc-2025) at 4°C overnight. The next day, 50 μl of equilibrated protein A/G-Agarose beads were added to each sample and rotated at 4°C for 4 hours. Then, 100 μl of the supernatant was saved for the flow-through sample, and the beads were washed two times with lysis buffer and then washed two times with lysis buffer containing 1 M urea. Then, the beads were boiled in tris-EDTA containing 1% SDS and 12% β-mercaptoethanol before RNA was extracted using a Direct-zol RNA Microprep kit (Zymo Research). The extracted RNA was then prepared for RT-qPCR analysis to examine *RB1, Actin*, and *GAPDH* expression.

### Coimmunoprecipitation

Cells were lysed in lysed in 1% NP-40 lysis buffer (50 mM Hepes, 150 mM NaCl, 1% NP-40, 1 mM EDTA with protease inhibitor mixture, and 1 mM phenylmethylsulfonyl fluoride). Lysates were incubated on ice for 30 min before clearing by centrifuging at 16,000*g* at 4°C for 20 min. Protein lysates were precleared with Protein A/G PLUS-Agarose beads (Santa Cruz Biotechnology, sc-2003) at 4°C for 1 hour. Then, 8% of cleared lysates were taken as input, the rest were incubated with either 5 μg of FLAG M2 antibody (MilliporeSigma, #F1804) or mouse IgG control (BioLegend, 400101) on a rotor at 4°C for 2 hours. Then, Protein A/G PLUS-Agarose beads (Santa Cruz Biotechnology, sc-2003) were added to each sample and incubated on a rotor at 4°C for 2 hours. Then, beads were washed three times in 1% NP-40 lysis buffer for 10 min each time at 4°C on a rotor. The immunoprecipitated proteins were then eluted in 1× sample buffer 2 (Invitrogen, 1981103) by boiling at 95°C for 10 min. Then, the samples were analyzed by immunoblotting.

For pulling down the ubiquitinated proteins, HEK293 cells were transfected with plasmids expressing Clover-3xFlag-RbWT, RbΔCDK, or Rb13EE using PEI, and then the cells were induced by Dox (1 μg/ml) for 24 hours. Then, cells were treated with bortezomib (1 μM) for 5 hours before collection. Cells were lysed in 1% NP-40 lysis buffer [50 mM Hepes, 150 mM NaCl, 1% NP-40, 1 mM EDTA with protease inhibitor mixture, and 1 mM phenylmethylsulfonyl fluoride (PMSF)], supplemented with 40 mM *N*-ethylmaleimide (Sigma-Aldrich, E3876) and 10 mM iodoacetamide (Sigma-Aldrich, I1149). Lysate was incubated on ice for 30 min and then centrifuged at 21,130*g* at 4°C for 20 min. Protein concentration was measured using a bicinchoninic acid protein assay kit (Pierce). For TUBE2 (LifeSensors, UM402) affinity captures, 1 mg of cell lysate was mixed with 20 μl of TUBE agarose and rotated at 4°C overnight. Beads were washed three times with lysis buffer, and the proteins were eluted in 1× sample buffer (Invitrogen, 1981103) by boiling at 95°C for 10 min. The samples were then analyzed by immunoblotting.

### Immunoblots

Cells were directly lysed with 1× NuPAGE LDS sample buffer (Invitrogen) and then incubated at 95°C for 10 min. Lysates were separated on NuPAGE 3 to 8% tris-acetate protein gels (Thermo Fisher Scientific) and transferred to nitrocellulose membranes. Membranes were then blocked with SuperBlock (tris-buffered saline) blocking buffer (Thermo Fisher Scientific) and incubated overnight at 4°C with primary antibodies in 3% BSA solution in PBS. The primary antibodies were detected using the fluorescently labeled secondary antibodies IRDye 680LT goat anti-mouse IgG (LI-COR, 926-68020) and IRDye 800CW goat anti-rabbit IgG (LI-COR, 926-32211). Membranes were imaged on a LI-COR Odyssey CLx and analyzed with LI-COR Image Studio software. The following primary antibodies were used: anti–β-actin (Sigma-Aldrich, A2103; 1:2000), anti-Rb (Santa Cruz Biotechnology, sc-74570; 1:500), anti-UBR5 (Millipore, ABE2863; 1:2000), anti-tubulin (Sigma-Aldrich, T5168; 1:2000), and anti–glyceraldehyde-3-phosphate dehydrogenase (GAPDH) (Sigma-Aldrich, AB2302; 1:2000).

### Image analysis

For live-cell imaging microscopy data, cell nuclei were segmented using the green fluorescent protein (GFP) channel from either the endogenously expressed Rb-3xFLAG-Clover-sfGFP or the inducibly overexpressed Clover-3xFlag-Rb variants. For fixed cell imaging microscopy data, cell nuclei were segmented using the DAPI DNA staining signal. Segmentation was performed using the Fiji plugin StarDist, which is a deep-learning tool for segmenting nuclei in images that are difficult to segment using thresholding-based methods. The total pixel intensities within the segmented masks in each channel were recorded, and each object’s background was subtracted on the basis of the median intensity of the image. Nuclear volume was used as a proxy for cell size and calculated as the nuclear area^3/2^. The tracking of live cells was done manually using the TrackMate plugin in Fiji. To determine the protein half-life, the degradation traces were fitted with an exponential decay function *N* = *N*_0_exp(−*kt*) so that the half-life *t*_1/2_ = (1/*k*)(ln2).

### RNA extraction and RT-qPCR

Total RNA was isolated using a Direct-zol RNA Miniprep kit (Zymo Research). For RT-qPCR, cDNA synthesis was performed with 1 μg of total RNA using an iScript Reverse Transcription Kit (Bio-Rad). qPCR reactions were made with the 2× SYBR Green Master Mix (Bio-Rad). Gene expression levels were measured using the ΔΔ*C*_t_ method.

### Mathematical model of Rb concentration dynamics

The mathematical model of Rb dynamics consists of two main equations. The first describes the time evolution of cell mass as $\frac{\mathrm{dM}}{\mathrm{dt}}=\gamma M^{\delta }$ , where *M* denotes the cell mass, δ < 1 quantifies the departure from pure exponential growth as measured in (*77*), and γ is a constant. The second equation describes the evolution of the amount of Rb in the cell as $\frac{d\text{Rb}}{\mathrm{dt}}=\alpha M^{\delta }-\beta \left(t\right)\;\text{Rb}$ , where α is an effective synthesis rate and β(*t*) is a time-varying degradation rate. Therefore, the amount of Rb synthesized at a given time is proportional to the cell’s growth rate. The distinctive element in this model is the time dependence of the degradation rate β(*t*), which reflects the cell cycle–dependent changes in Rb stability. It is encoded as a step change such that β(*t*) = β_0_ as long as the cell is in G_1_ phase and that β(*t*) = εβ_0_, ε < 1, when the cell is in S-G_2_-M phase. To compare the relative contributions of cell cycle–dependent synthesis and degradation changes, we also consider the case where β is a constant and α increases slightly in a step change at the G_1_-S transition. The cell goes through the G_1_-S transition when the concentration of Rb in the cell crosses a threshold value set a priori. The S-G_2_-M phase is represented as a timer in the model. The model is integrated in time with an Euler forward method. From arbitrary initial conditions, the model is run through a “burn-in” period until it reaches a limit cycle described by the input parameters. The model is available on GitHub (https://github.com/LucasFuentesValenzuela/RB_model).

### Genomic analysis of patients with breast cancer

We extracted the copy number data from Pereira *et al.* (*78*) (allele-specific copy number analysis of tumors) and used the IC subtypes reported in the study of Rueda *et al.* (*59*). We performed survival analysis using Cox’s proportional hazard models using R package survival (version 3.5-7) and corrected for key clinical covariates as described in (*59*) (e.g., age, grade, tumor size, lymph node, and ER status). We generated Kaplan-Meier and forest plots using the R package survminer (version 0.4.9).

### Statistical analysis

The data in most figure panels reflect multiple biological replicate experiments performed on different days. The mouse experiments used mice derived from different litters. For comparison between groups, we generally conducted unpaired two-tailed Student’s *t* tests. Statistical significance is displayed as *P < 0.05 or **P < 0.01 unless specified otherwise.

## Supplementary Materials

### The PDF file includes:

Figs. S1 to S18 Legends for tables S1 to S36 Uncropped Western blots
