# Supplementary material for: The G1-S transition is promoted by Rb degradation via the E3 ligase UBR5
Source: Sci Adv. 2024 Oct 23;10(43):eadq6858. doi: 10.1126/sciadv.adq6858 (PMC11498223; doi:10.1126/sciadv.adq6858)

Science Advances
AAAS

Supplementary Materials for

The G_1-S transition is promoted by Rb degradation via the E3 ligase UBR5

Shuyuan Zhang *et al.*

Corresponding author: Jan M. Skotheim, skotheim@stanford.edu

Sci. Adv. **10**, eadq6858 (2024)
DOI: 10.1126/sciadv.adq6858

The PDF file includes:

Figs. S1 to S18
Legends for tables S1 to S36
Uncropped Western blots

Other Supplementary Material for this manuscript includes the following:

Tables S1 to S36

Supplementary figures and captions

Fig. S1

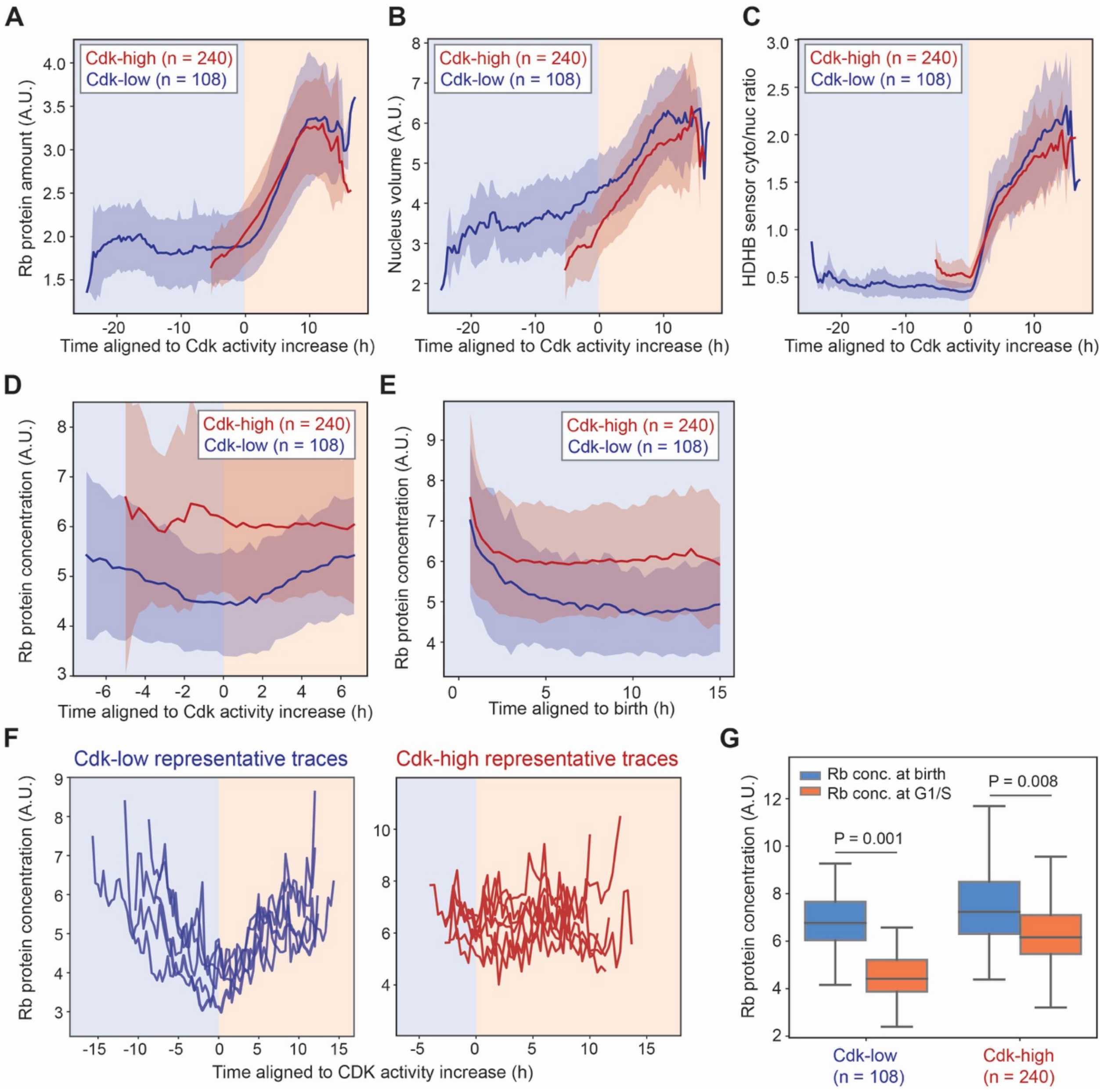

Fig. S1. Rb concentration continuously decreases in early G1 phase in Cdk-low cells.
A-E. Live-cell imaging of HMEC-hTERT1 cells expressing endogenously tagged *RB1-3xFLAG-Clover-sfGFP* and the HDHB CDK sensor [1]. Cdk-high and Cdk-low cells were grouped as in Fig. 1C. Average traces for the total amount of Rb-3xFLAG-Clover-sfGFP protein (A), nuclear volume (B), the cytoplasmic-to-nuclear intensity ratio of the HDHB sensor (C), and the protein concentration of Rb-3xFLAG-Clover-sfGFP (D, E) are plotted. Traces are aligned by the inflection point of the cytoplasm-to-nuclear ratio of the HDHB sensor, which indicates the initial rise of CDK activity (A-C), or aligned at birth (E). Shaded region denotes the standard deviation. **F**. Representative single cell traces showing the Rb concentration dynamics of Cdk-low and Cdk-high cells. Cells were aligned to the G1/S transition. **G**. Comparing Rb concentrations at birth or at the G1/S transition (Cdk activation point) in Cdk-low and Cdk-high cells. The statistics are comparing across 3 independent experiments.

Fig. S2

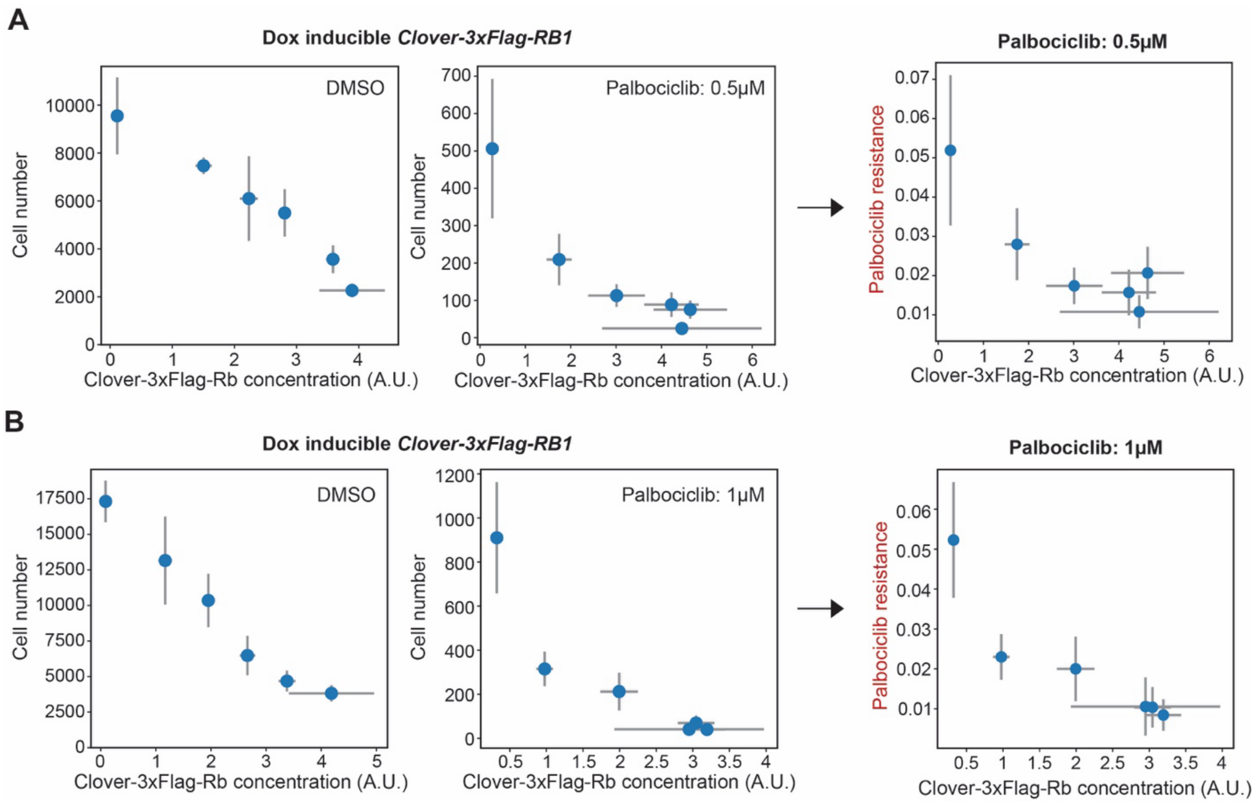

Fig. S2. Rb overexpression increases the sensitivity of cells to CDK4/6 inhibition
A-B. Cell number and normalized cell number of HMEC cells treated with 0.5μM (A) or 1μM (B) Palbociclib for 72 hours. Cells were plated in different doses of doxycycline to induce exogenous Clover-3xFlag-Rb. Drug treatment started the next day and lasted for 72 hours. Then, cells were fixed and the cell number in each well was measured. Normalized cell number is the cell number under Palbociclib treatment divided by the cell number under DMSO treatment. N = 3 biological replicates and the error bars indicate standard deviations.

Fig. S3

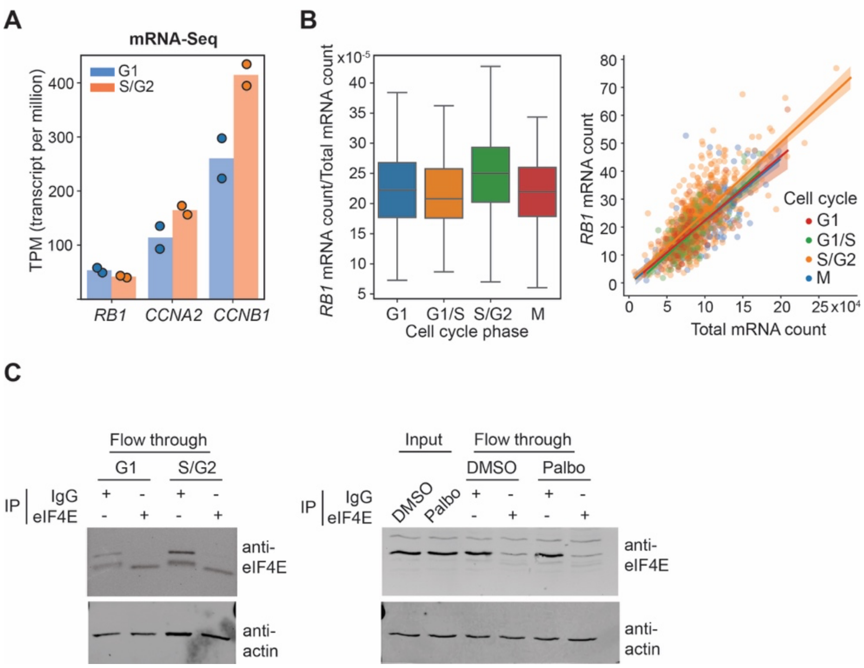

Fig. S3. The synthesis rate of *RB1* is relatively constant through the cell cycle.
A. mRNA-Seq (n = 2) measurements for *RB1* mRNA concentration in G1 and S/G2 HMEC cells sorted using a FUCCI marker. **B.** Analysis of published MERFISH data(*35*) for *RB1* mRNA concentrations. (Left panel) the *RB1* mRNA concentration in different cell cycle phases classified using the cell cycle dependent genes identified from the paper. *RB1* mRNA concentration is calculated by dividing the *RB1* mRNA count by the total mRNA count. (Right panel) mRNA count of *RB1* is proportional to the total mRNA count in different cell cycle phases. **C.** Western blot of eIF4E. RIP (RNA binding protein immunoprecipitation) assay for pulling down eIF4E was performed on G1 and S/G2 cell populations or DMSO and Palbociclib (1μM) treated cells.

Fig. S4

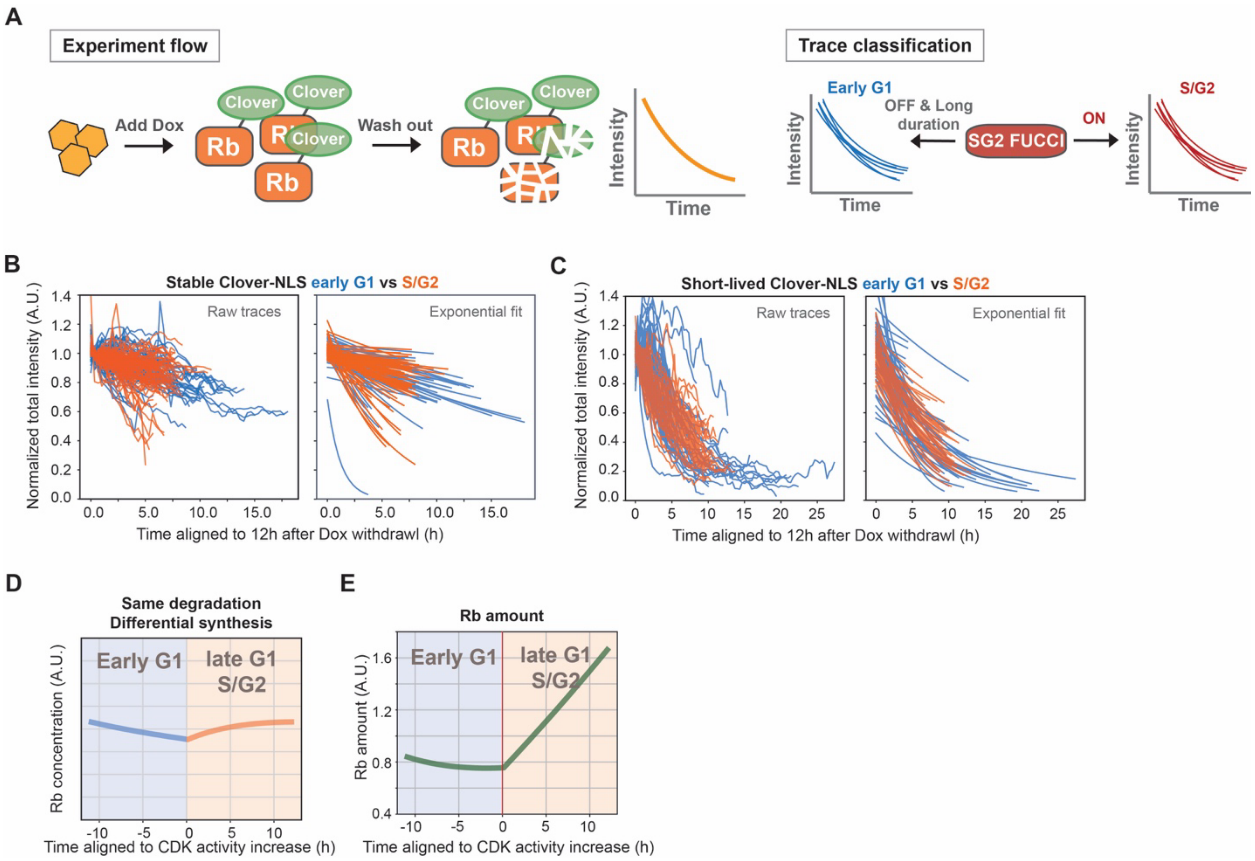

Fig. S4. Rb is degraded in early G1 phase.

A. Schematic of the live-cell imaging approach for measuring the protein degradation rate in different cell cycle phases. **B.** Degradation traces for stable Clover-NLS and the corresponding exponential fits (n=88). **C.** Degradation traces of short-lived Clover-NLS and the corresponding exponential fits (n=57). **D-E**. Mathematical model of Rb concentration and protein amount dynamics during cell cycle progression. (D) Rb concentration dynamics assuming that its degradation rate does not change, but its synthesis rate increases by 20% at the G1/S transition as measured in Fig. 2A. (E) Total Rb protein amount dynamics assuming that its degradation rate decreases by 80% at the G1/S transition as measured by live imaging (Fig. 2C) and its synthesis rate does not change. Related to Fig. 2E.

Fig. S5

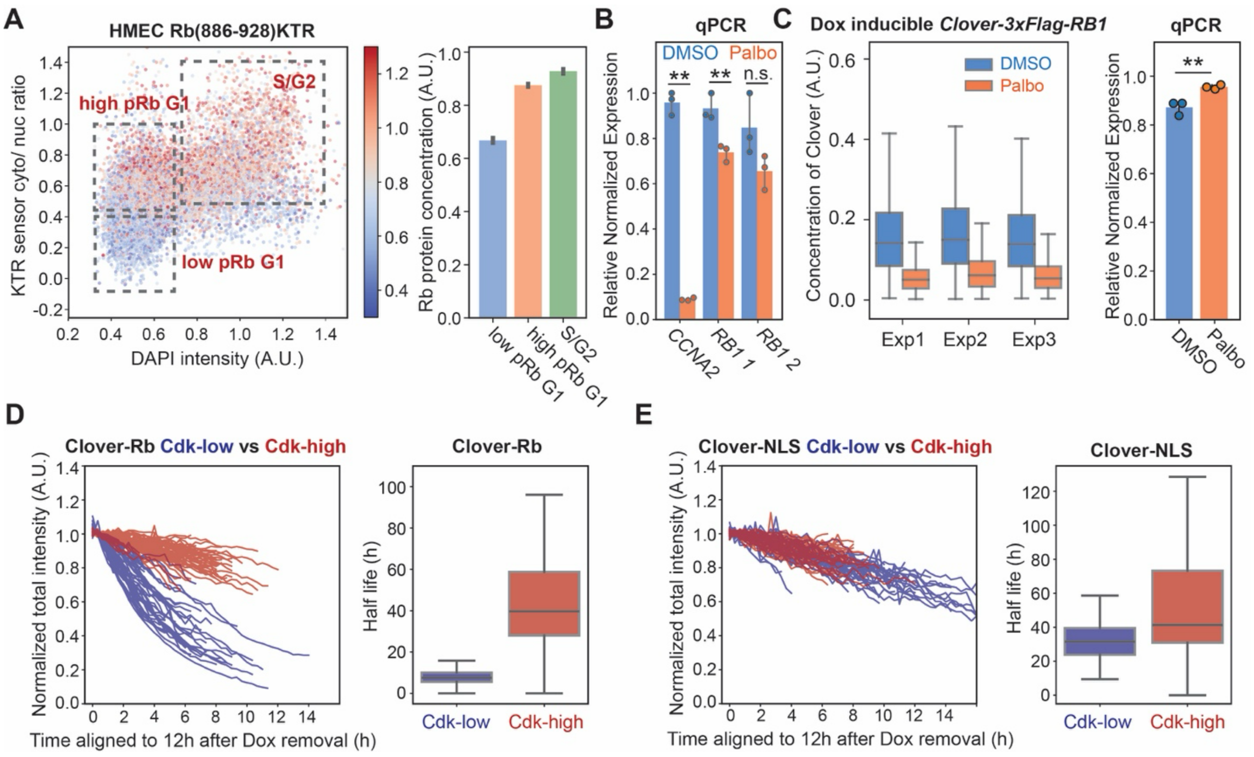

Fig. S5. Rb is stabilized by Cdk mediated phosphorylation

A. Rb concentration in cell populations with different CDK activities. HMEC cells expressing the Rb(886-928) KTR sensor (*37*) were stained with an Rb antibody. (Left panel) KTR sensor cytoplasmic-to-nucleus intensity ratio is plotted against DNA content (DAPI intensity). Dot color indicates Rb concentration. (Right panel) quantification of Rb concentrations in populations with different CDK activities as indicated in regions in the left panel. Bars denote 95% confidence interval for the mean. **B.** *RB1* qPCR measurement of mRNA from cells treated with DMSO or Palbociclib (1µM, 24 hours); related to Fig. 2B. n=3 and dots denote biological replicates. Bars denote standard deviation. **C.** Protein and mRNA concentrations of Clover-3xFlag-Rb. Cells expressing Clover-3xFlag-Rb (Dox induced for 48 hours) were treated with DMSO or Palbocliclib (1µM) for 24 hours. The protein concentration of Clover-3xFlag-Rb (left panel) was measured by flow cytometry. The concentration was defined as Clover fluorescent intensity divided by side scatter area (SSC-A). The mRNA concentration of Clover-3xFlag-Rb (right panel) was measured using qPCR with primers targeting Clover. **D-E.** (Left panel) The degradation traces of Clover-3xFlag-Rb protein (D) or Clover-NLS (E) after Dox withdrawal. The traces were classified into Cdk-low and Cdk-high cells based on the HDHB Cdk sensor. (Right panel) Distribution of half-lives estimated from the exponential fit. Box plot indicates 5th, 25th, median, 75th and 95th percentiles.

Fig. S6

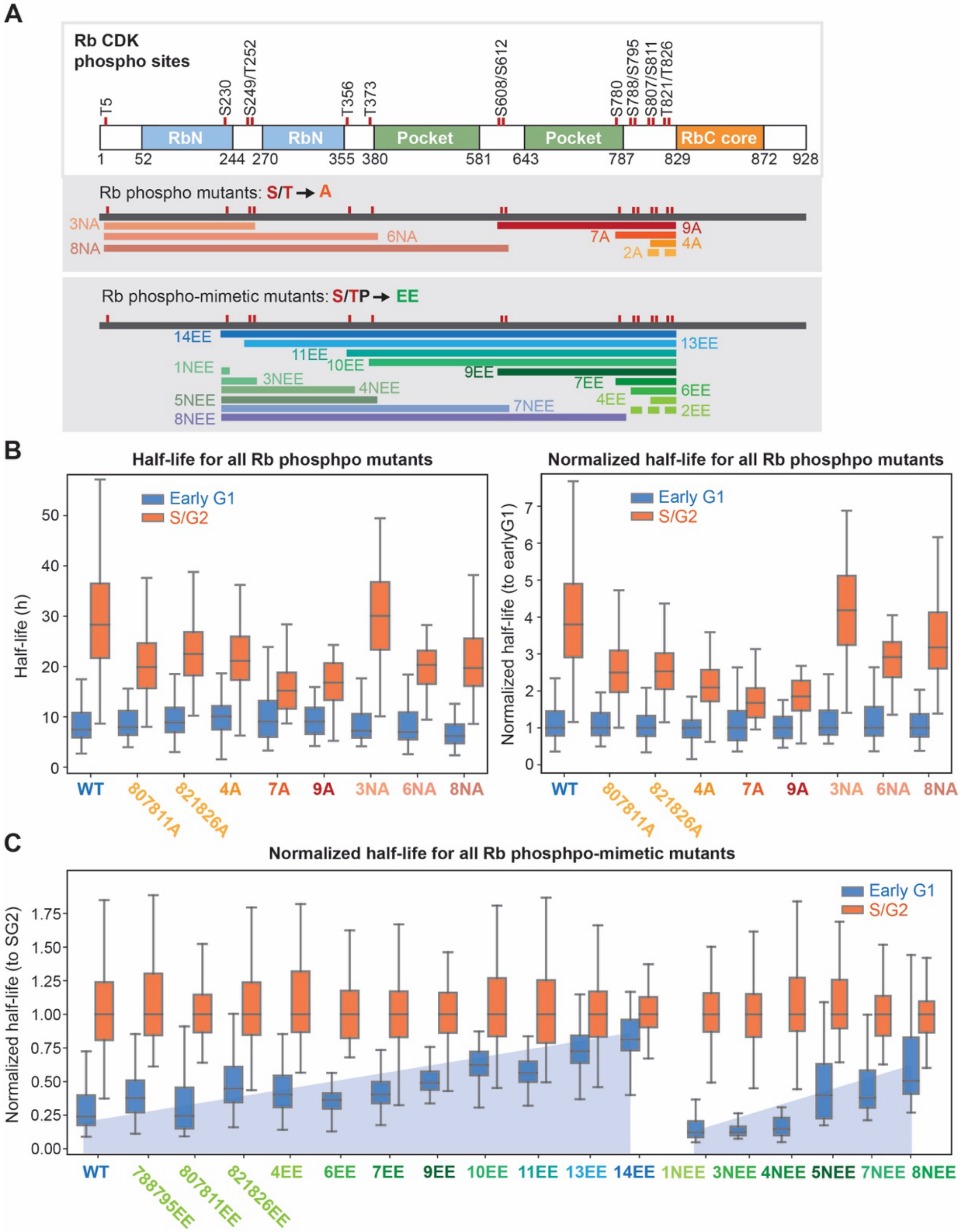
A
Rb CDK phospho sites
T5
S230
S249/T252
T356
T373
S608/S612
S780
S788/S795
S807/S811
T821/T826
RbN
RbN
Pocket
Pocket
RbC core
1 52 244 270 355 380 581 643 787 829 872 928
Rb phospho mutants: S/T → A
3NA
6NA
8NA
9A
7A
4A
2A
Rb phospho-mimetic mutants: S/TP → EE
14EE
13EE
11EE
10EE
9EE
1NEE
3NEE
4NEE
7EE
6EE
5NEE
4EE
7NEE
2EE
8NEE
B
Half-life for all Rb phosphpo mutants
Normalized half-life for all Rb phosphpo mutants
Early G1
S/G2
Half-life (h)
Normalized half-life (to earlyG1)
WT
807811A
821826A
4A
7A
9A
3NA
6NA
8NA
C
Normalized half-life for all Rb phosphpo-mimetic mutants
Normalized half-life (to SG2)
WT
788795EE
807811EE
821826EE
4EE
6EE
7EE
9EE
10EE
11EE
13EE
14EE
1NEE
3NEE
4NEE
5NEE
7NEE
8NEE

Fig. S6. Half-life measurements of Rb phospho-mutants and phospho-mimetic mutants
A. Schematics of the CDK sites on Rb that were mutated in the Rb phospho-mutants and the Rb phospho-mimetic mutants used in this study. **B.** The half-lives (left) and normalized half-lives (right) for all the Rb phospho-mutants measured using the live-cell imaging approach. The normalized half-lives were calculated by normalizing to the half-life in early G1 for each Rb protein variant. **C.** Normalized half-lives of all the Rb phospho-mimetic mutants measured using the live-cell imaging approach. Related to Fig. 3E, F. Here, the normalized half-lives were calculated by normalizing to the half-life in S/G2 for each Rb protein variant.

Fig. S7

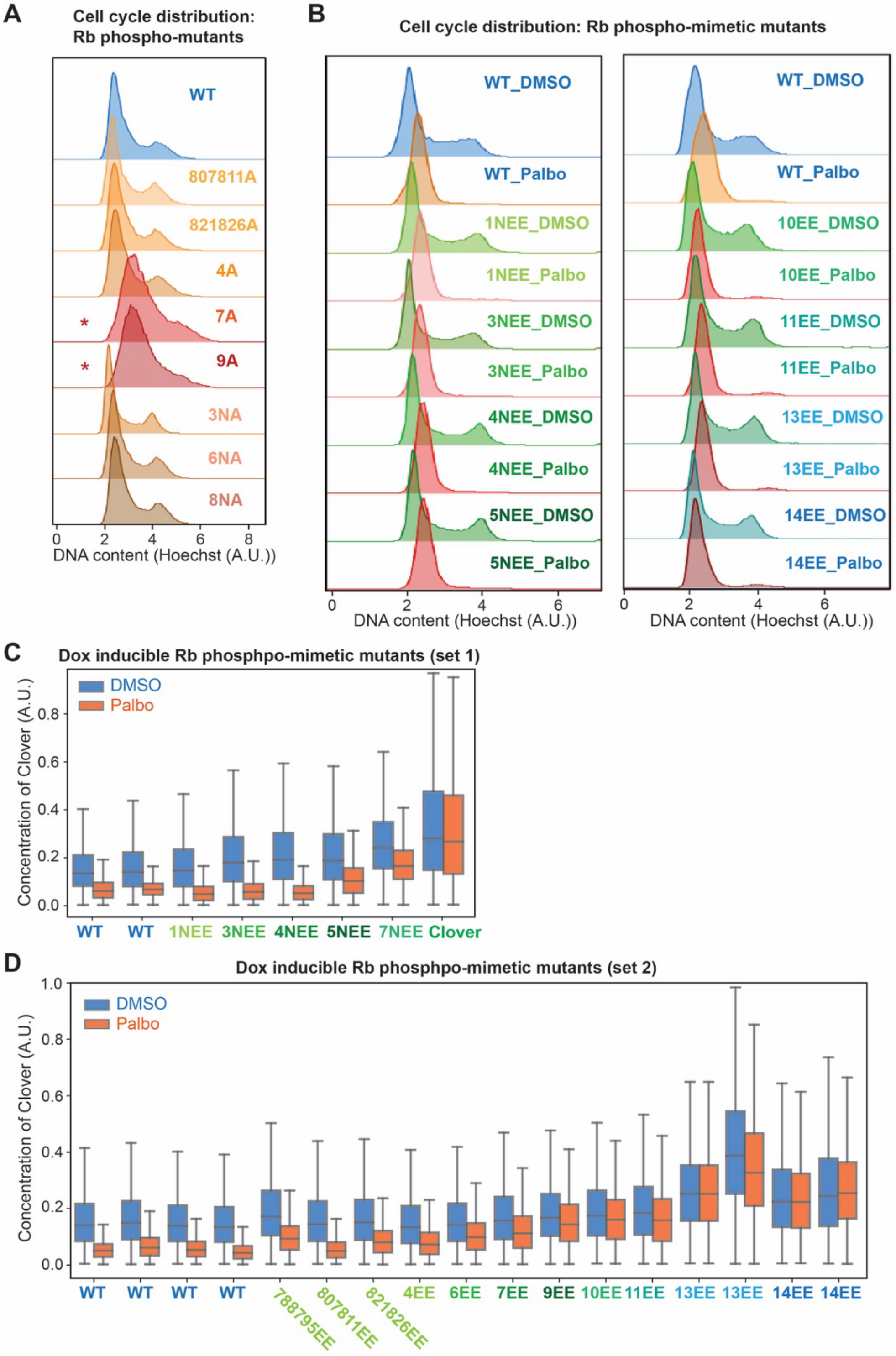
A
Cell cycle distribution: Rb phospho-mutants
WT
807811A
821826A
4A
7A
9A
3NA
6NA
8NA
DNA content (Hoechst (A.U.))
B
Cell cycle distribution: Rb phospho-mimetic mutants
WT_DMSO
WT_Palbo
1NEE_DMSO
1NEE_Palbo
3NEE_DMSO
3NEE_Palbo
4NEE_DMSO
4NEE_Palbo
5NEE_DMSO
5NEE_Palbo
10EE_DMSO
10EE_Palbo
11EE_DMSO
11EE_Palbo
13EE_DMSO
13EE_Palbo
14EE_DMSO
14EE_Palbo
DNA content (Hoechst (A.U.))
C
Dox inducible Rb phosphpo-mimetic mutants (set 1)
DMSO
Palbo
Concentration of Clover (A.U.)
WT WT 1NEE 3NEE 4NEE 5NEE 7NEE Clover
D
Dox inducible Rb phosphpo-mimetic mutants (set 2)
DMSO
Palbo
Concentration of Clover (A.U.)
WT WT WT WT 788795EE 807811EE 821826EE 4EE 6EE 7EE 9EE 10EE 11EE 13EE 13EE 14EE 14EE

Fig. S7. Cdk phospho-site mutations affect Rb stability

A. Cell cycle distribution for cells expressing different Rb phospho-mutants. Cells expressing Clover-3xFlag-Rb phospho-mutants (Dox induced for 48 hours) were incubated with Hoechst for 30 minutes to stain DNA, and then analyzed by flow cytometry. Red stars indicate that cells expressing Rb7A and Rb9A are arrested in G1. **B.** Cell cycle distribution for cells expressing Rb phospho-mimetic mutants. Cells expressing Clover-3xFlag-Rb phospho-mimetic mutants (Dox induced for 48 hours) were treated with DMSO or Palbocliclib (1μM) for 24 hours. Then the cells were incubated with Hoechst for 30 minutes to stain DNA, and analyzed by flow cytometry. **C-D**. The concentrations of Clover-3xFlag-Rb phospho-mimetic mutants (induced by Dox for 48 hours) after 24 hours of DMSO or Palbociclib (1μM) treatment, as measured by flow cytometry. (**C**) shows the mutations made from the N-terminus of Rb, and (**D**) shows the mutations made from the C-terminus of Rb.

Fig. S8

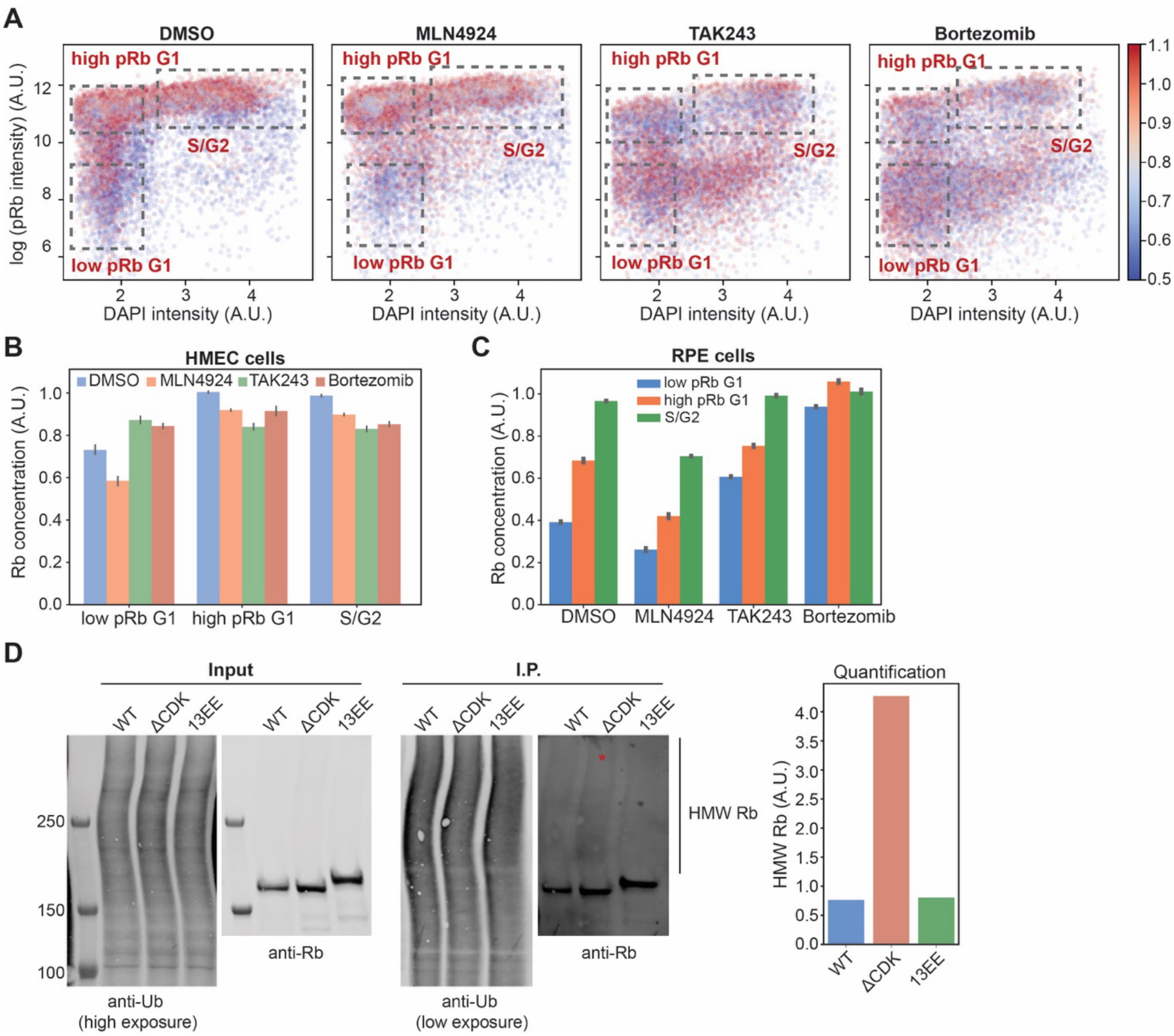

Fig. S8. Un/hypo-phosphorylated Rb is degraded through the Ubiquitin-proteasome system
A. HMEC cells were treated with the indicated drugs for 5 hours, fixed, and stained with phospho-Rb (S807/811) and Rb antibodies. The Rb concentration is calculated by dividing total Rb intensity by nuclear area$^{3/2}$. The dot color indicates Rb concentration. **B**. Rb concentration in different phospho-Rb populations of HMEC cells shown in A. **C.** Rb concentration in different phospho-Rb populations of RPE-1 cells after treatments with the indicated drugs for 6 hours, as measured by flow cytometry. Rb concentration was calculated by dividing the Rb total intensity with the side scatter area (SSC-A). **D.** HEK293 cells were transfected with plasmids expressing Clover-3xFlag-RbWT, RbΔCDK or Rb13EE (induced by 1µg/ml Dox) for 24 hours. Then, cells were treated with Bortezomib (1µM) for 5 hours before collection. After lysis, the ubiquitinated proteins were pulled down using TUBE beads. The samples were then detected for Rb and Ubiquitin using immunoblot. Right panel shows the quantification of the HMW Rb intensity of the I.P. samples.

Fig. S9

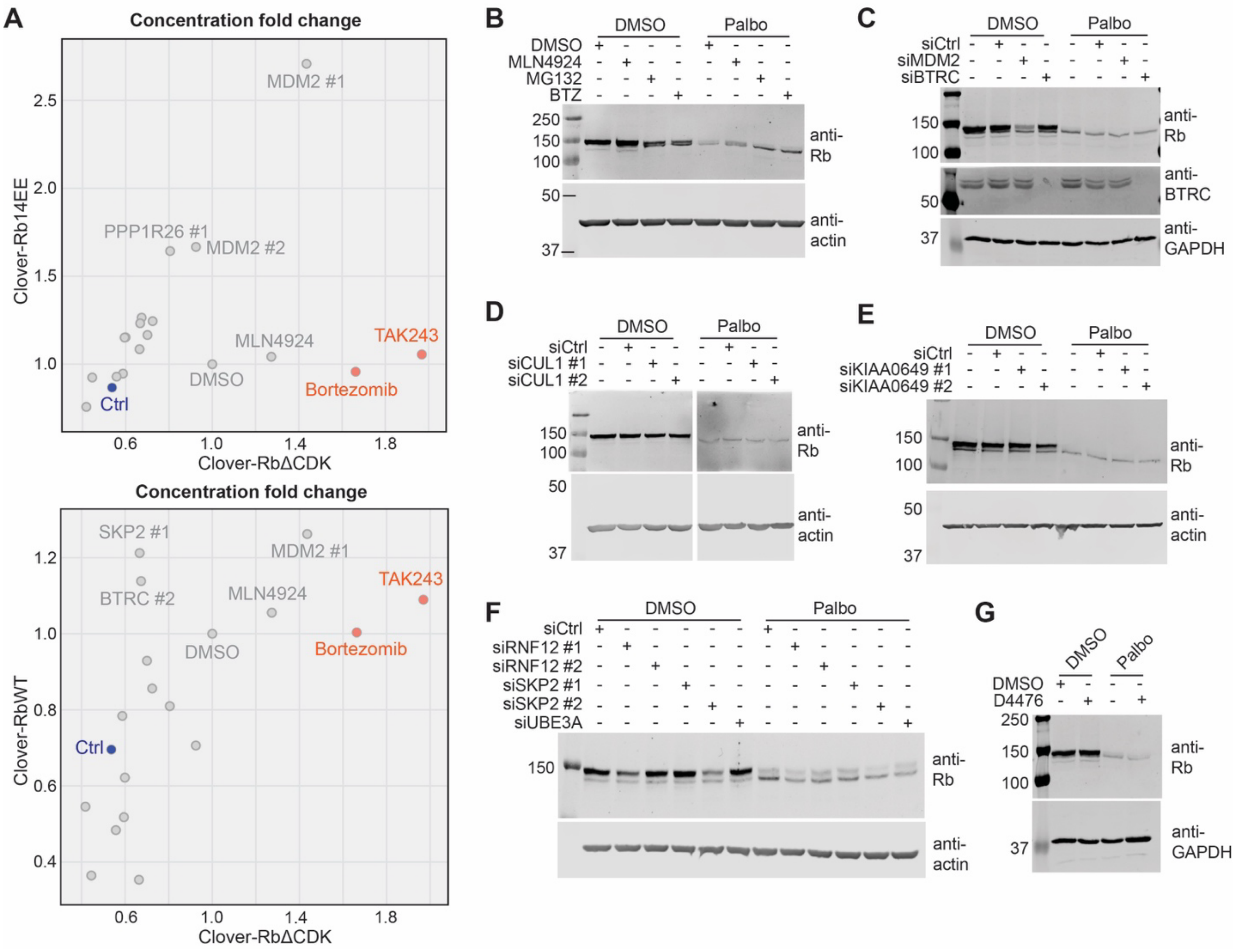

Fig. S9. Examination of E3 ligases reported to target Rb

A. Comparison of concentration fold change after siRNA treatment. HMEC cells expressing Clover-3xFlag-RbΔCDK, Clover-3xFlag-RbWT, or Clover-3xFlag-Rb14EE (Dox induced for 48 hours) were transfected with siRNAs against the published E3 ligase genes for 48 hours. Then, the cells were fixed and imaged. The concentration fold change is calculated by dividing the Clover concentration of the treatment well by the concentration in the DMSO treated well. Red dots indicate the positive controls, *i.e.*, TAK243 and Bortezomib treatments (5 hours of treatment), and the blue dot indicates the siCtrl treated sample. **B-F**. The published E3 ligase genes for Rb were also examined by western blot analysis. HMEC cells were transfected with the corresponding siRNAs for 24 hours, and then treated with DMSO or Palbociclib (1µM) for 24 hours. Cells were then harvested for western blotting with Rb antibodies. The lower band in the Rb western blots represents un/hypo-phosphorylated Rb. **G.** The effect of CK1 on Rb half-life was examined using western blot analysis. Cells were treated with DMSO vs Palbociclib (1µM) and DMSO vs D4476 (25µM CK1 inhibitor), and then the cells were lysed for western blotting.

Fig. S10

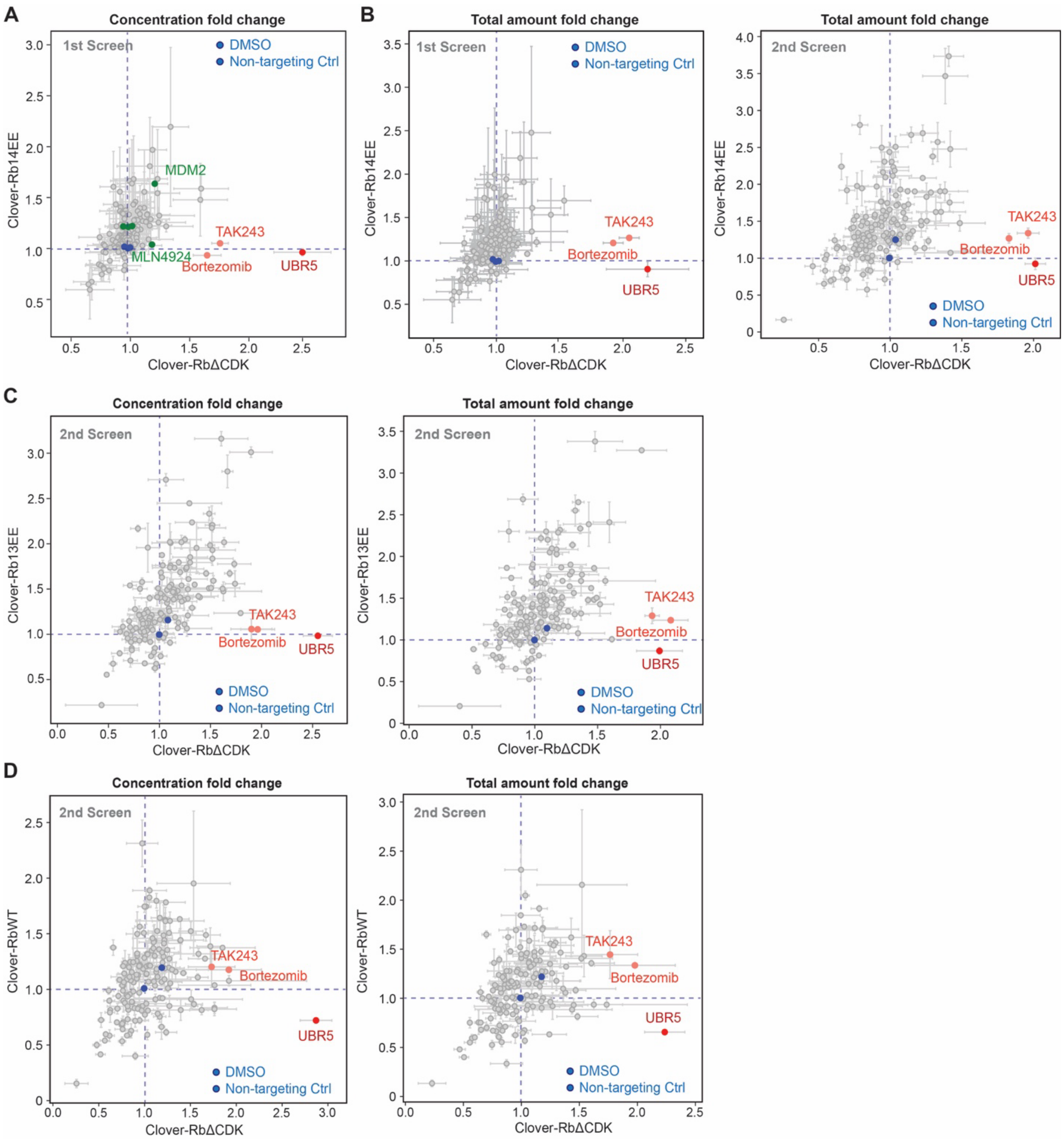
A
Concentration fold change
1st Screen
DMSO
Non-targeting Ctrl
MDM2
MLN4924
TAK243
Bortezomib
UBR5
Clover-Rb14EE
Clover-RbΔCDK
B
Total amount fold change
1st Screen
2nd Screen
C
Clover-Rb13EE
D
Clover-RbWT

Fig. S10. UBR5 is identified as a candidate E3 ligase targeting Rb in siRNA screens
A. Same plot as Fig. 4D (1st siRNA screen), except that the green dots are added to indicate the previously reported E3 genes for Rb. **B.** The total amount fold changes of Clover-3xFlag-RbΔCDK and Clover-3xFlag-Rb14EE are plotted. Related to Fig. 4D, E. **C.** Concentration fold changes and total amount fold changes of Clover-3xFlag-RbΔCDK and Clover-3xFlag-Rb13EE from the 2nd siRNA screen. n = 2 biological replicates. **D.** Concentration fold changes and total amount fold changes of Clover-3xFlag-RbΔCDK and Clover-3xFlag-RbWT from the 2nd siRNA screen. n = 2 biological replicates.

Fig. S11

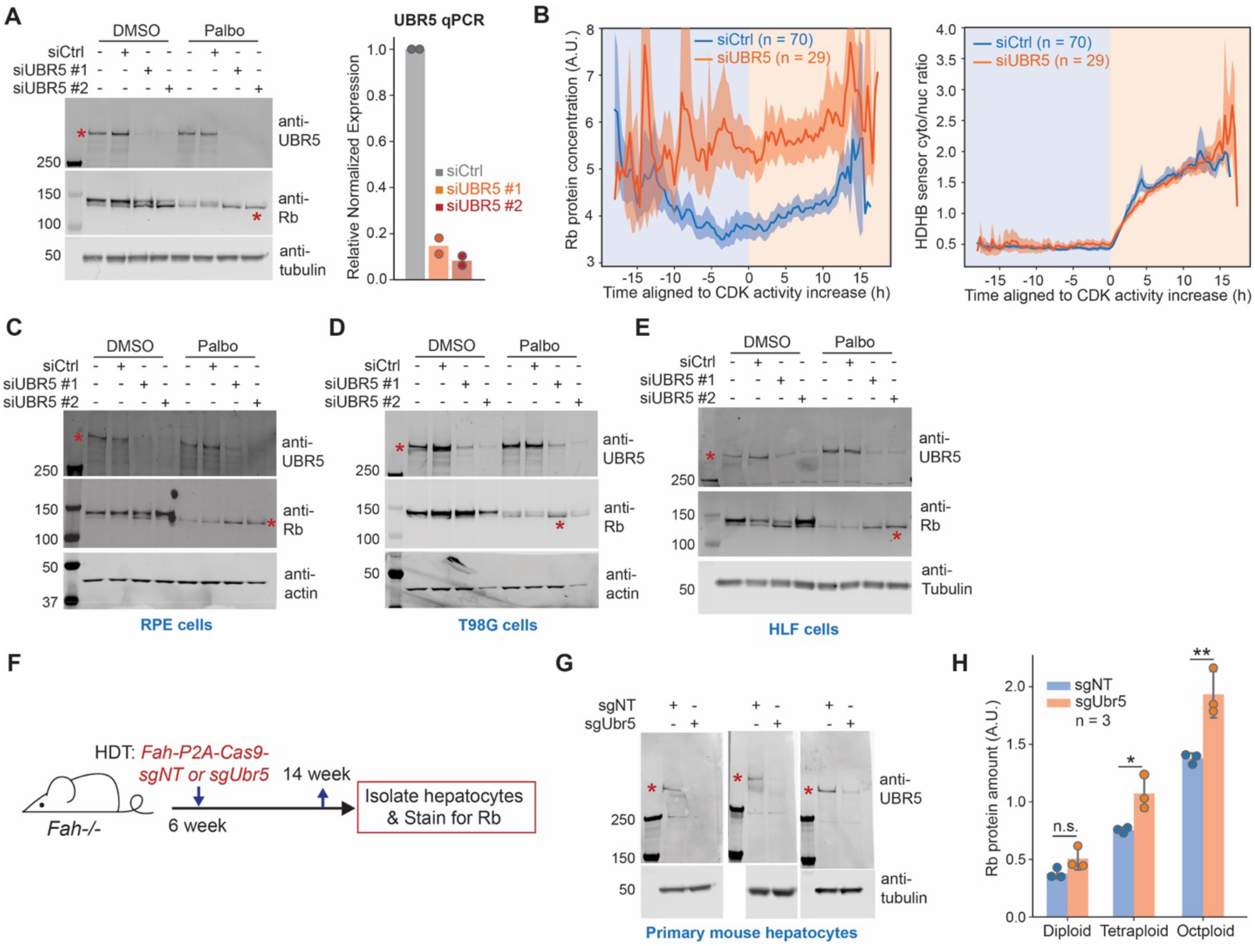

A
DMSO
Palbo
siCtrl
siUBR5 #1
siUBR5 #2
anti-UBR5
anti-Rb
anti-tubulin
UBR5 qPCR
Relative Normalized Expression
siCtrl
siUBR5 #1
siUBR5 #2
B
Rb protein concentration (A.U.)
siCtrl (n = 70)
siUBR5 (n = 29)
Time aligned to CDK activity increase (h)
HDHB sensor cyto/nuc ratio
C
RPE cells
anti-actin
D
T98G cells
E
HLF cells
anti-Tubulin
F
HDT: Fah-P2A-Cas9-sgNT or sgUbr5
6 week
14 week
Isolate hepatocytes & Stain for Rb
Fah-/-
G
sgNT
sgUbr5
Primary mouse hepatocytes
H
sgNT
sgUbr5
n = 3
Rb protein amount (A.U.)
n.s.
Diploid
Tetraploid
Octploid

Fig. S11. UBR5 is responsible for Rb degradation in multiple cell types
A. Left panel: Immunoblot of Rb after UBR5 knockdown by siRNA. Cells were treated with siRNAs for 24 hours, and then treated with DMSO or Palbociclib (1μM) for 24 hours before harvest. The lower band in the Rb blot indicates the un/hypo-phosphorylated Rb (marked by red star). Right panel: qPCR validation of UBR5 knockdown efficiency. HMEC cells were transfected with siRNA for 48 hours. **B.** Live imaging validation of Rb concentration dynamics during cell cycle progression. HMEC cells expressing an endogenously tagged *RB1-3xFLAG-Clover-sfGFP* and an HDHB CDK sensor [1] were transfected with UBR5 siRNA for 24 hours and then imaged. Average traces for Rb-3xFLAG-Clover-sfGFP concentration (left panel) or the cytoplasmic-to-nuclear intensity ratio of the HDHB sensor (right panel) are plotted; solid lines indicate mean values for each time point and shaded regions indicate the standard deviation of the mean. Traces are aligned using the HDHB sensor to identify the time that CDK activity begins to increase. **C-E.** Western blot validation of the role of UBR5 in Rb degradation in multiple cell lines (**C.** RPE cells, **D.** T98G cells, **E.** HLF cells). Cells were transfected with siRNAs against UBR5 or Ctrl siRNA. 24 hours later, DMSO or Palbociclib (1μM) were added for another 24 hours. Then, cells were lysed for western blot analysis. The lower band of the Rb blot is marked by a red star, which represents the un/hypo-phosphorylated Rb. **F**. Experimental flow of the *Fah-/-* mouse liver model. 6-week old *Fah-/-* mice were hydrodynamically transfected with plasmids carrying an *Fah-P2A-Cas9-sgNT* transposon or an *Fah-P2A-Cas9-sgUbr5* transposon, together with a transposase plasmid. 8 weeks later, hepatocytes were isolated from the mice for downstream analysis. **G.** Western blot validation of UBR5 knockout efficiency in primary hepatocytes. *Fah-/-* mice were injected with transposons carrying *Cas9-sgNT* or *Cas9-sgUbr5*. Primary hepatocytes were isolated from the mice, plated, and then lysed for western blot analysis. n = 3 pairs of mice. **H.** Total Rb amount in the low-phospho-Rb population of the primary hepatocytes isolated from mice receiving *Fah-P2A-Cas9-sgNT* or *Fah-P2A-Cas9-sgUbr5* transposons. n = 3 pairs of mice. Error bar indicates standard deviation. Related to Fig. 5D.

Fig. S12

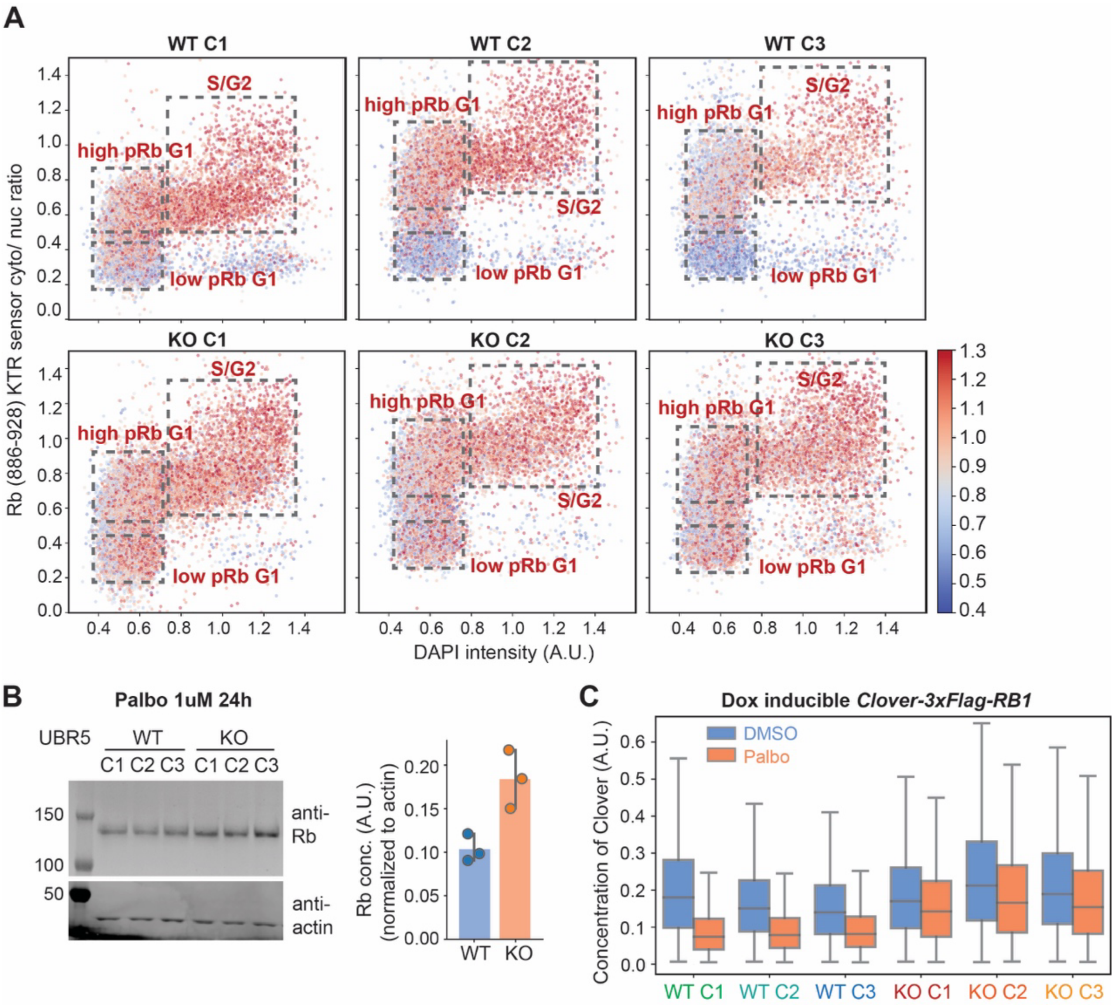

Fig. S12. Rb concentration is increased in the low-phospho-Rb populations of *UBR5 KO* clonal cell lines

A. Rb staining of *UBR5 WT* or *KO* clonal cell lines expressing the Rb (886-928) KTR sensor (*37*). The cytoplasmic-to-nucleus intensity ratio of the KTR sensor is plotted against DNA content (DAPI). The dot color indicates the Rb concentration. Related to Fig. 4B. **B.** Western blot analysis of Rb concentration after Palbociclib treatment in UBR5 clonal cell lines. 6 UBR5 clonal cell lines (3 WT, 3 KO) were treated with DMSO or Palbociclib (1µM) for 24 hours, and then lysed for western blotting. Quantification of Rb concentration (Rb band intensity normalized to the actin band intensity) is shown on the right. Dots denote individual clones and error bars denote the standard deviation. **C.** Flow cytometry analysis of Clover-3xFlag-Rb concentration after Palbociclib treatment. 6 UBR5 clonal cell lines (3 WT, 3 KO) expressing Clover-3xFlag-RbWT (Dox induced for 48 hours) were treated with DMSO or Palbociclib (1µM) for 24 hours, and then cells were analyzed using flow cytometry. The concentration of Clover-3xFlag-RbWT was defined as Clover fluorescent intensity divided by side scatter area (SSC-A).

Fig. S13

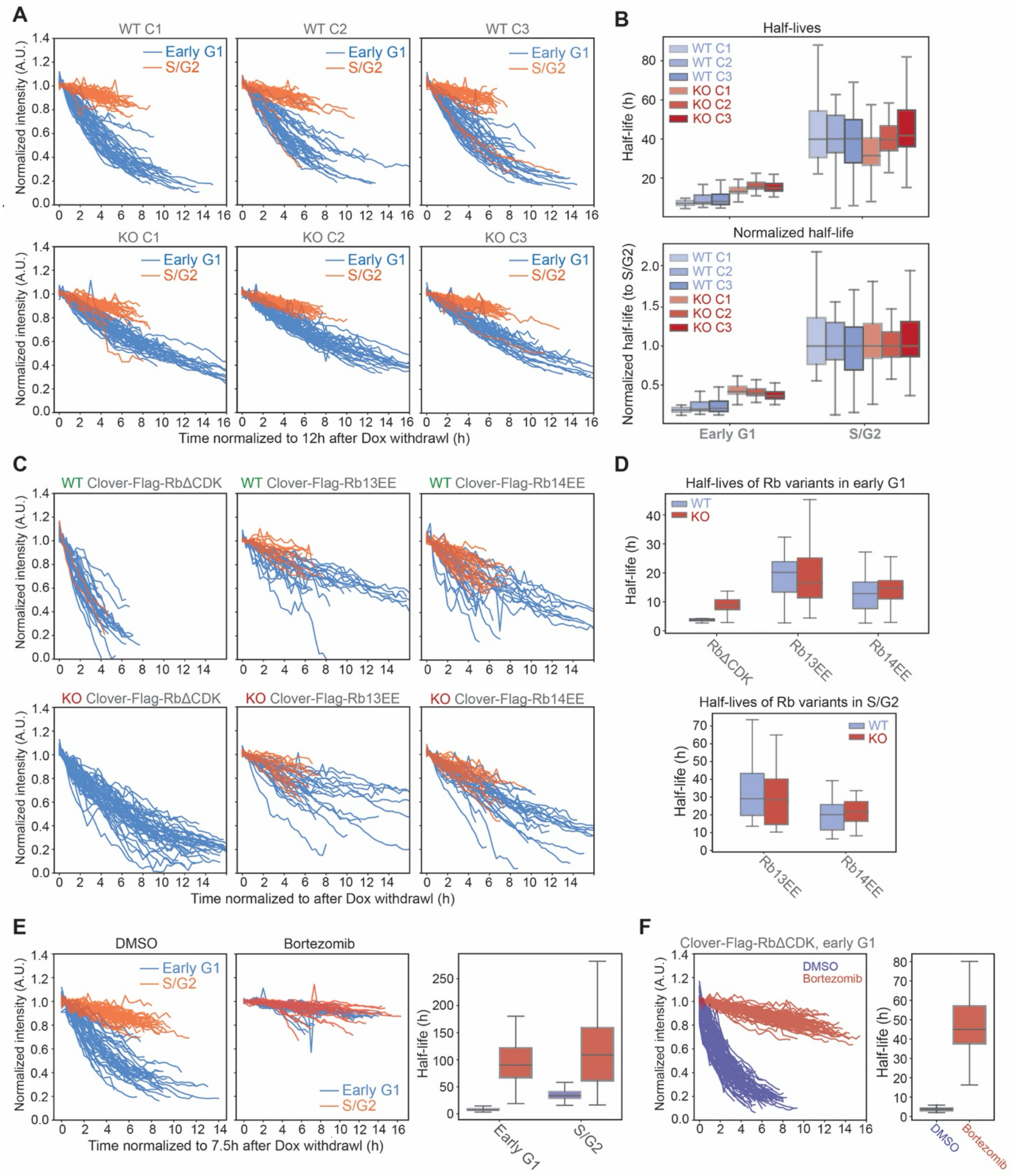
A
WT C1
WT C2
WT C3
KO C1
KO C2
KO C3
Early G1
S/G2
Normalized intensity (A.U.)
Time normalized to 12h after Dox withdrawl (h)
B
Half-lives
Half-life (h)
Normalized half-life
Normalized half-life (to S/G2)
Early G1
S/G2
C
WT Clover-Flag-RbΔCDK
WT Clover-Flag-Rb13EE
WT Clover-Flag-Rb14EE
KO Clover-Flag-RbΔCDK
KO Clover-Flag-Rb13EE
KO Clover-Flag-Rb14EE
Time normalized to after Dox withdrawl (h)
D
Half-lives of Rb variants in early G1
WT
KO
RbΔCDK
Rb13EE
Rb14EE
Half-lives of Rb variants in S/G2
E
DMSO
Bortezomib
Time normalized to 7.5h after Dox withdrawl (h)
F
Clover-Flag-RbΔCDK, early G1

Fig. S13. Un-phosphorylated Rb is stabilized in *UBR5 KO* cells

A. The degradation traces of Clover-3xFlag-RbWT protein after Dox withdrawal in 6 UBR5 clonal cell lines (3 WT, 3 KO). The traces were classified into early G1 phase or S/G2 phase based on the FUCCI cell cycle reporter. **B.** The half-life distributions (upper panel) and normalized half-lives (lower panel, normalized to the median of S/G2 half-life) calculated from the degradation traces shown in (**A**). **C**. The degradation traces of Clover-3xFlag-RbΔCDK, Clover-3xFlag-Rb13EE, and Clover-3xFlag-Rb14EE protein after Dox withdrawal in UBR5 WT and KO cell lines. The traces were classified into early G1 phase or S/G2 phase based on a FUCCI cell cycle reporter. **D**. The half-life distributions of the Rb variants in (C) in early G1 phase (upper panel) or in S/G phase (lower panel) calculated from the degradation traces shown in (C). **E**. Left two panels: The degradation traces of Clover-3xFlag-RbWT protein after Dox withdrawal under DMSO or Bortezomib (1uM) treatment. The traces were classified into early G1 phase or S/G2 phase based on the FUCCI cell cycle reporter prior to Bortezomib treatment. Right panel: The half-life distributions calculated from the degradation traces. **F.** Left panel: The degradation traces of Clover-3xFlag-RbΔCDK protein after Dox withdrawal under DMSO or Bortezomib (1uM) treatment. Only the early G1 traces are shown here because the cells are arrested in G1 due to RbΔCDK expression. Right panel: The half-life distributions calculated from the degradation traces.

Fig. S14

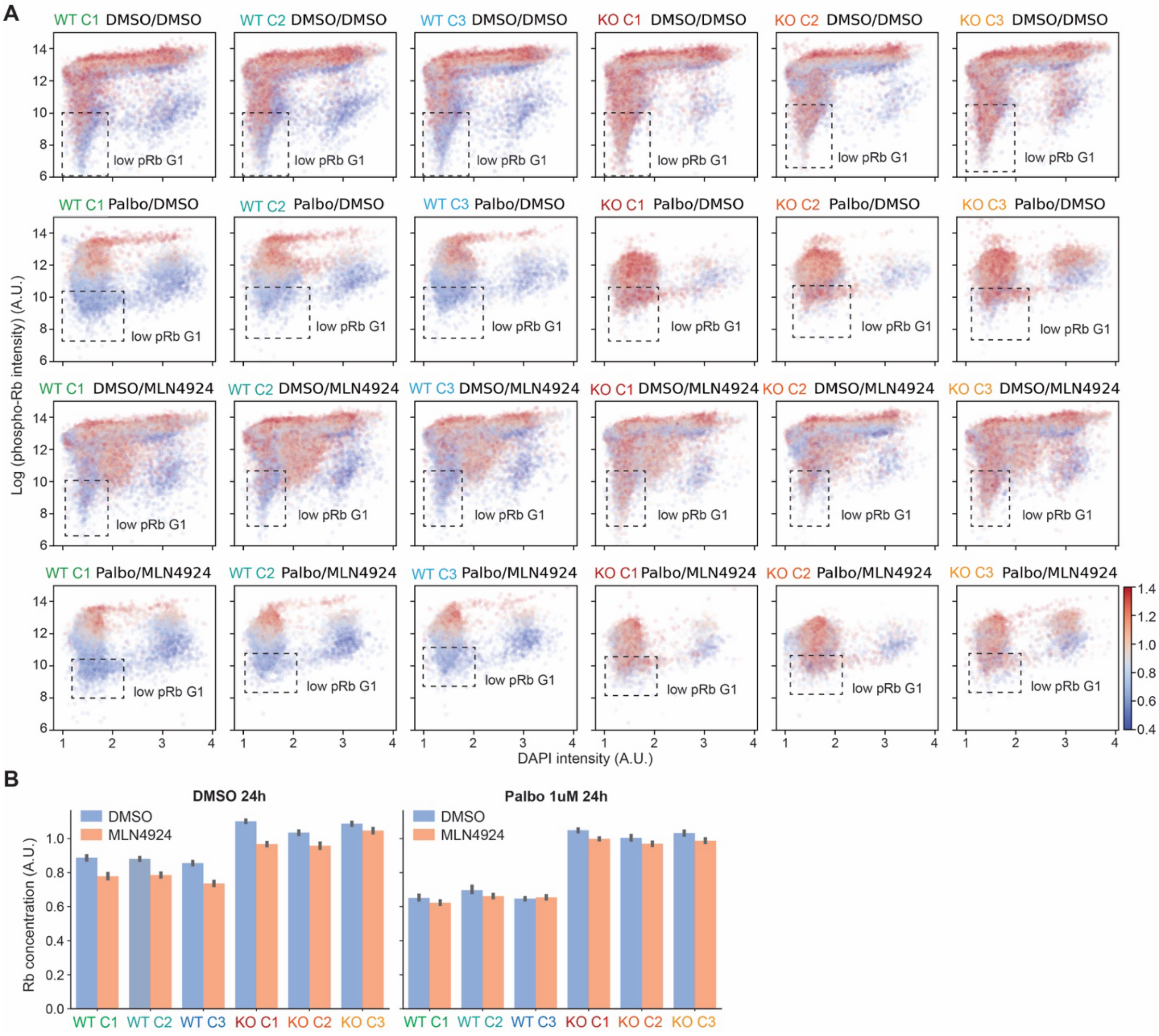

Fig. S14. The Rb concentration is increased in the low-phospho-Rb populations of *UBR5 KO* clonal cell lines

A. phospho-Rb concentration is plotted against DNA content in UBR5 clonal cell lines. 6 UBR5 clonal cell lines (3 WT, 3 KO) were treated with DMSO or Palbociclib (1µM) for 24 hours, and then the cells were treated with DMSO or MLN4924 (1µM) for 5 hours before fixation. Cells were stained with phospho-Rb (S807/811) and Rb antibodies, followed by imaging. The dot color indicates the Rb concentration. The dashed line box indicates the low pRb G1 population that has low concentration of phospho-Rb. Plot titles indicate the cell genotype and the treatments administered. *E.g.*, Palbo/DMSO indicates that 1 µM Palbociclib was administered before DMSO. **B.** Quantification of the Rb concentration in the low pRb G1 populations of cells in (A). Error bar indicates the 95% confidence interval of the mean.

Fig. S15

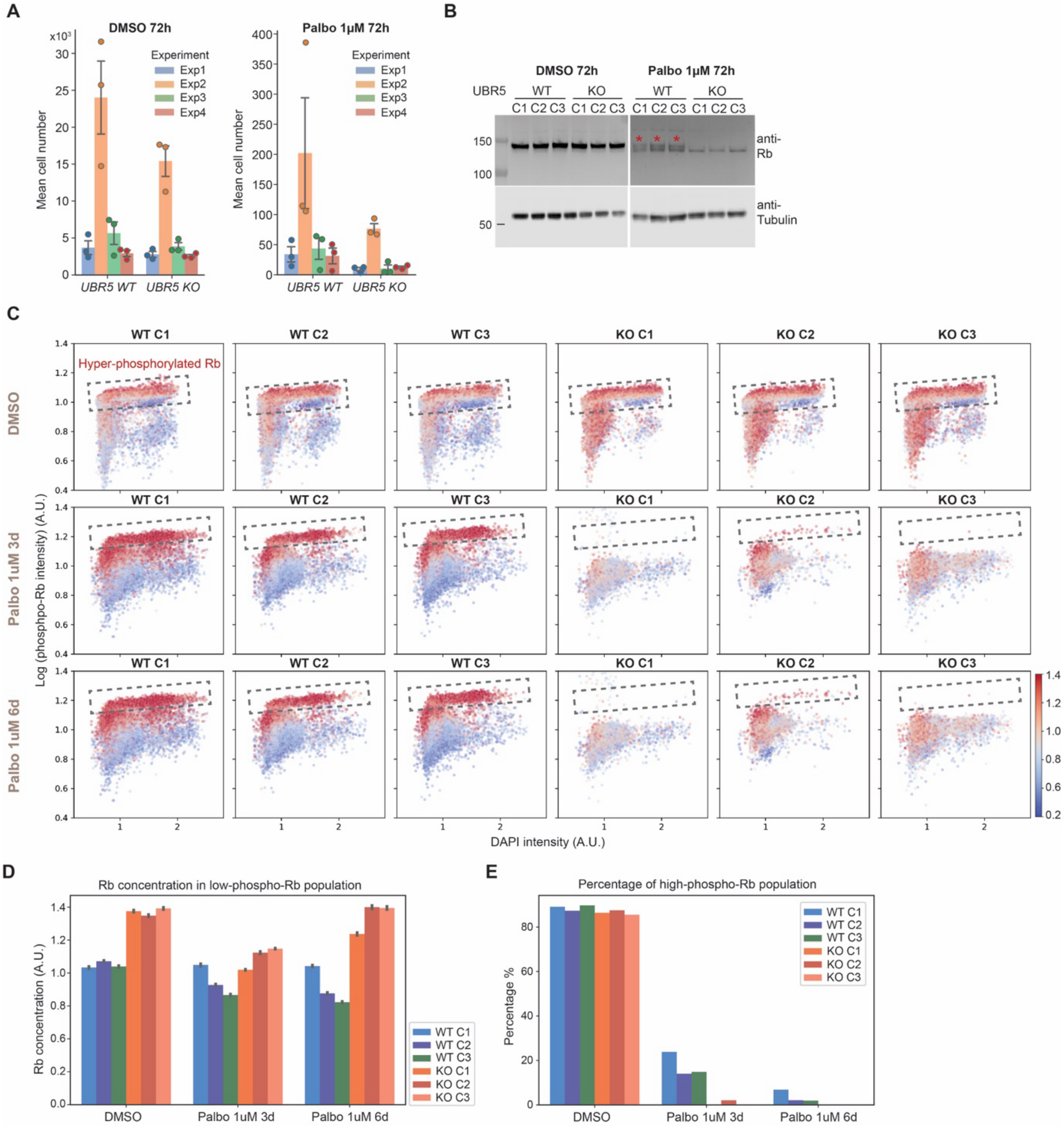
A
DMSO 72h
Palbo 1μM 72h
Mean cell number
Experiment
Exp1
Exp2
Exp3
Exp4
UBR5 WT
UBR5 KO
B
DMSO 72h
Palbo 1μM 72h
UBR5
WT
KO
C1 C2 C3
anti-Rb
anti-Tubulin
C
Hyper-phosphorylated Rb
WT C1
WT C2
WT C3
KO C1
KO C2
KO C3
DMSO
Palbo 1uM 3d
Palbo 1uM 6d
Log (phosphpo-Rb intensity) (A.U.)
DAPI intensity (A.U.)
D
Rb concentration in low-phospho-Rb population
Rb concentration (A.U.)
E
Percentage of high-phospho-Rb population
Percentage %

Fig. S15. ***UBR5 KO*** **cells are sensitized to Palbociclib treatment**

A. The absolute cell number counts for 6 UBR5 clonal cell lines (3 WT, 3 KO) after DMSO or Palbociclib (1μM) treatment for 72 hours; related to Fig. 6E. **B-C.** Western blot (**B**) and immunostaining (**C**) analysis of phospho-Rb (S807/811) in 6 UBR5 clonal cell lines (3 WT, 3 KO) after DMSO or Palbociclib (1μM) treatment for 3 days or 6 days. The hyper-phosphorylated Rb population is marked using red stars (the upper band in (B)) or dashed lined boxes (in (C)). **D**. Quantification of the Rb concentration in the low pRb G1 populations of cells in (C). Error bar indicates the 95% confidence interval of the mean. **E**. Quantification of the percentage of hyper-phosphorylated Rb population of cells in (C).

Fig. S16

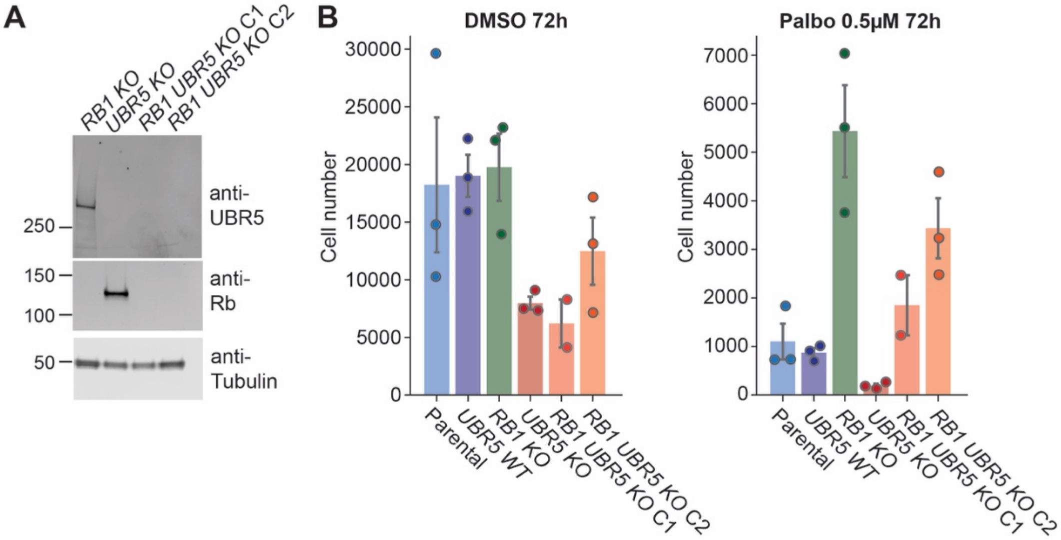

Fig. S16. The increased sensitivity to Palbocilib treatment in *UBR5 KO* cells depends on Rb. **A.** Western blot validation of UBR5 and Rb double KO cell lines. **B**. The absolute cell number count for wild-type, *UBR5 KO*, *RB1 KO*, and *UBR5 RB1 KO* cells after DMSO or Palbociclib (0.5μM) treatment for 72 hours; related to Fig. 4F. Bars denote the standard deviation of the mean.

Fig. S17

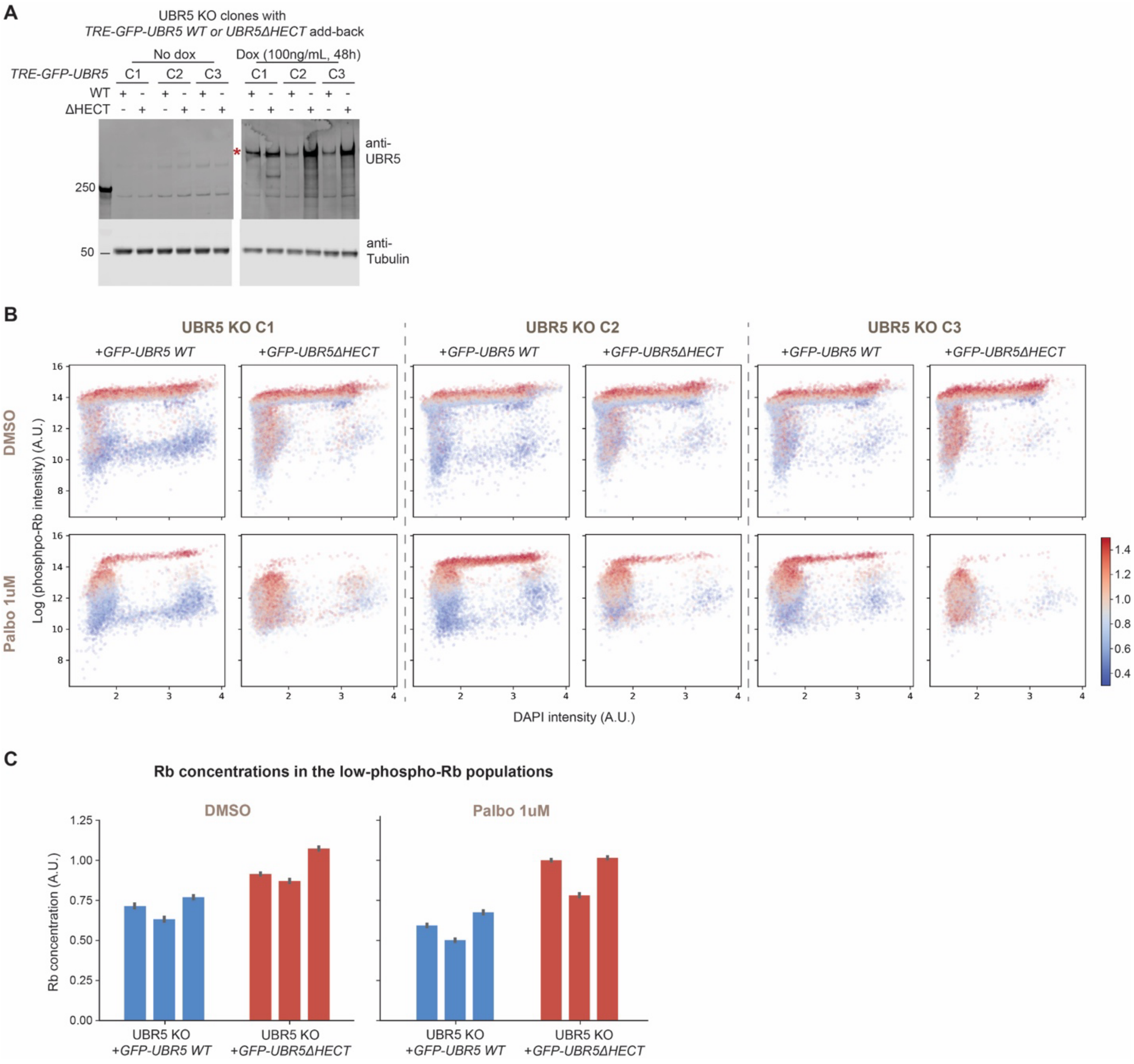

Fig. S17. Adding back WT UBR5, but not mutant UBR5, decreases Rb concentration in *UBR5 KO* cells

A. Western blot validation of *UBR5 KO* cell lines (3 KO clones) with UBR5WT or UBR5ΔHECT add-back using the Dox inducible system. Cells were induced with Dox (100ng/ml) for 48 hours and then lysed for western blot analysis. No-dox and Dox treated samples were run on separate gels. **B**. immunostaining analysis of phospho-Rb (S807/811) in 3 *UBR5 KO* clonal cell lines with UBR5WT or UBR5ΔHECT add-back (as described in A) after DMSO or Palbociclib (1μM) treatment for 24 hours. The dot color indicates the Rb concentration. **C**. Quantification of the Rb concentration in the low-pRb G1 populations of cells in (B). Error bar indicates the 95% confidence interval of the mean.

Fig. S18

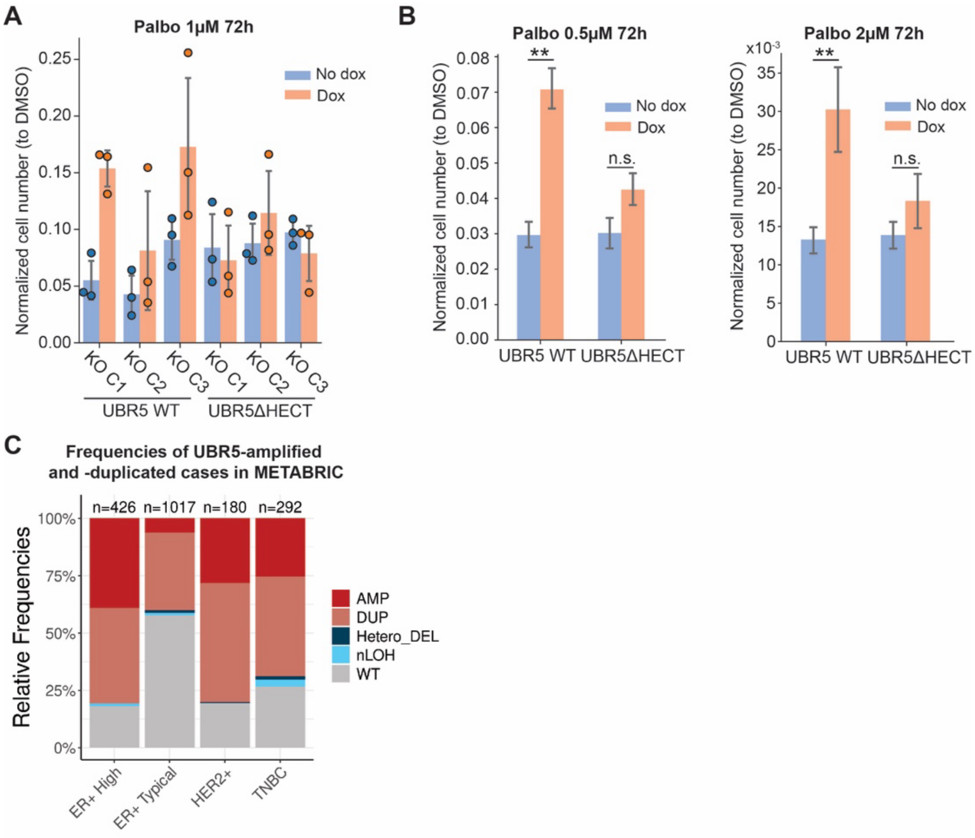

Fig. S18. Adding back WT URB5 rescues the Palbociclib-sensitivity phenotype.
A. Normalized cell number of *UBR5 KO* cells with UBR5 WT or UBR5ΔHECT addback after Palbociclib (0.5µM) treatment for 72 hours (related to Fig. 4G). **B.** Normalized cell number of *UBR5 KO* cells with UBR5 WT or UBR5ΔHECT addback after Palbociclib (0.5µM or 2µM) treatment for 72 hours. The results are the average of 3 *UBR5 KO* clonal cell lines with different UBR5 addback and 3 biological replicates. All error bars denote standard deviations. **C.** Frequencies of *UBR5*-amplification and -duplication in the METABRIC dataset across different breast cancer subtypes. The number of tumors within each group is indicated on the top of each bar. AMP = amplification with total copy number ≥ 6; DUP = duplication with total copy number ≥ 3; Hetero_DEL = heterozygous deletion; nLOH = neutral loss-of-heterozygosity.

Table S1 (Separate file)

Gene names and siRNA sequences of the siRNA libraries.

Table S2 (Separate file)

Quantifications of the live imaging experiments on HMEC-hTERT1 cells expressing endogenously tagged *RB1-3xFLAG-Clover-sfGFP* and the HDHB CDK sensor. Related to Fig. 1 and Fig. S1.

Table S3 (Separate file)

Quantifications of the live imaging experiment measuring the half-lives of Clover-3xFlag-Rb protein in early G1 and S/G2 phases. Related to Fig. 2C.

Table S4 (Separate file)

Quantifications of the live imaging experiment measuring the half-lives of Rb-3xFlag-Clover protein in early G1 and S/G2 phases. Related to Fig. 2D.

Table S5 (Separate file)

Quantifications of the live imaging experiment measuring the half-lives of short-lived Clover-NLS protein in early G1 and S/G2 phases. Related to Fig. 2D and Fig. S4C.

Table S6 (Separate file)

Quantifications of the live imaging experiment measuring the half-lives of stable Clover-NLS protein in early G1 and S/G2 phases. Related to Fig. 2D and Fig. S4B.

Table S7 (Separate file)

Quantifications of the live imaging experiment measuring the half-lives of Clover-Rb8NA protein in early G1 and S/G2 phases. Related to Fig. 3C.

Table S8 (Separate file)

Quantifications of the live imaging experiment measuring the half-lives of Clover-Rb9A protein in early G1 and S/G2 phases. Related to Fig. 3C.

Table S9 (Separate file)

Summary of the half-lives and normalized half-lives for all the Rb phospho-mutants measured using the live-cell imaging approach. Related to Fig. S6B.

Table S10 (Separate file)

Quantifications of the live imaging experiment measuring the half-lives of Clover-Rb9EE protein in early G1 and S/G2 phases. Related to Fig. 3D.

Table S11 (Separate file)

Quantifications of the live imaging experiment measuring the half-lives of Clover-Rb13EE protein in early G1 and S/G2 phases. Related to Fig. 3D.

Table S12 (Separate file)

Summary of the half-lives and normalized half-lives for all the Rb phosphor-mimetic mutants measured using the live-cell imaging approach. Related to Fig. 3E, F, and Fig. S6C.

Table S13 (Separate file)

Summary of the results of 2 siRNA screens. The table shows the fold changes of protein concentration, total protein amount, cell area, and cell number of Clover-3xFlag-RbΔCDK and Clover-3xFlag-Rb14EE for each siRNA treated samples. The fold change is calculated by dividing the treatment well by the non-treated well. n = 4 biological replicates for the first screen and n = 3 biological replicates for the second screen. Related to Fig. 4D and Fig. S10A, B.

Table S14 (Separate file)

Summary of the 2nd siRNA screen results comparing RbΔCDK vs RbWT and RbΔCDK vs Rb13EE. The table shows the fold changes of protein concentration, total protein amount, cell area, and cell number of Clover-3xFlag-RbΔCDK and Clover-3xFlag-RbWT or Rb13EE for each siRNA treated samples. The fold change is calculated by dividing the treatment well by the non-treated well. n = 3 biological replicates. Related to Fig. S10C, D.

Table S15 (Separate file)

Quantifications of the live imaging experiment measuring the half-lives of Clover-3xFlag-Rb protein in early G1 and S/G2 phases under the treatment of siCtrl. Related to Fig. 5B.

Table S16 (Separate file)

Quantifications of the live imaging experiment measuring the half-lives of Clover-3xFlag-Rb protein in early G1 and S/G2 phases under the treatment of siUBR5 #1. Related to Fig. 5B.

Table S17 (Separate file)

Quantifications of the live imaging experiment measuring the half-lives of Clover-3xFlag-Rb protein in early G1 and S/G2 phases under the treatment of siUBR5 #2. Related to Fig. 5B.

Table S18 (Separate file)

Quantifications of the live imaging experiment measuring the half-lives of Clover-3xFlag-Rb protein in early G1 and S/G2 phases in UBR5 WT clone #1. Related to Fig. 6C and Fig. S13A, B.

Table S19 (Separate file)

Quantifications of the live imaging experiment measuring the half-lives of Clover-3xFlag-Rb protein in early G1 and S/G2 phases in UBR5 WT clone #2. Related to Fig. 6C and Fig. S13A, B.

Table S20 (Separate file)

Quantifications of the live imaging experiment measuring the half-lives of Clover-3xFlag-Rb protein in early G1 and S/G2 phases in UBR5 WT clone #3. Related to Fig. 6C and Fig. S13A, B.

Table S21 (Separate file)
Quantifications of the live imaging experiment measuring the half-lives of Clover-3xFlag-Rb protein in early G1 and S/G2 phases in UBR5 KO clone#1. Related to Fig. 6C and Fig. S13A, B.

Table S22 (Separate file)
Quantifications of the live imaging experiment measuring the half-lives of Clover-3xFlag-Rb protein in early G1 and S/G2 phases in UBR5 KO clone#2. Related to Fig. 6C and Fig. S13A, B.

Table S23 (Separate file)
Quantifications of the live imaging experiment measuring the half-lives of Clover-3xFlag-Rb protein in early G1 and S/G2 phases in UBR5 KO clone#3. Related to Fig. 6C and Fig. S13A, B.

Table S24 (Separate file)
Quantifications of the live imaging experiment measuring the half-lives of Clover-3xFlag-RbΔCDK protein in early G1 and S/G2 phases in UBR5 WT clone. Related to Fig. 6D and Fig. S13C, D.

Table S25 (Separate file)
Quantifications of the live imaging experiment measuring the half-lives of Clover-3xFlag-Rb13EE protein in early G1 and S/G2 phases in UBR5 WT clone. Related to Fig. 6D and Fig. S13C, D.

Table S26 (Separate file)
Quantifications of the live imaging experiment measuring the half-lives of Clover-3xFlag-Rb14EE protein in early G1 and S/G2 phases in UBR5 WT clone. Related to Fig. 6D and Fig. S13C, D.

Table S27 (Separate file)
Quantifications of the live imaging experiment measuring the half-lives of Clover-3xFlag-RbΔCDK protein in early G1 and S/G2 phases in UBR5 KO clone. Related to Fig. 6D and Fig. S13C, D.

Table S28 (Separate file)
Quantifications of the live imaging experiment measuring the half-lives of Clover-3xFlag-Rb13EE protein in early G1 and S/G2 phases in UBR5 KO clone. Related to Fig. 6D and Fig. S13C, D.

Table S29 (Separate file)
Quantifications of the live imaging experiment measuring the half-lives of Clover-3xFlag-Rb14EE protein in early G1 and S/G2 phases in UBR5 KO clone. Related to Fig. 6D and Fig. S13C, D.

Table S30 (Separate file)
Quantifications of the live imaging experiment measuring the half-lives of Clover-3xFlag-Rb protein in Cdk-low and Cdk-high cells. Related to Fig. S5D.

Table S31 (Separate file)
Quantifications of the live imaging experiment measuring the half-lives of Clover-NLS protein in Cdk-low and Cdk-high cells. Related to Fig. S5E.

Table S32 (Separate file)
Quantifications of the live imaging experiments on HMEC-hTERT1 cells expressing endogenously tagged *RB1-3xFLAG-Clover-sfGFP* and the HDHB CDK sensor, treated with siCtrl or siUBR5. Related to Fig. S11B.

Table S33 (Separate file)
Quantifications of the live imaging experiment measuring the half-lives of Clover-3xFlag-Rb protein in early G1 and S/G2 phases under DMSO treatment. Related to Fig. S13E.

Table S34 (Separate file)
Quantifications of the live imaging experiment measuring the half-lives of Clover-3xFlag-Rb protein in early G1 and S/G2 phases under Bortezomib treatment. Related to Fig. S13E.

Table S35 (Separate file)
Quantifications of the live imaging experiment measuring the half-lives of Clover-3xFlag-RbΔCDK protein in early G1 and S/G2 phases under DMSO treatment. Related to Fig. S13F.

Table S36 (Separate file)
Quantifications of the live imaging experiment measuring the half-lives of Clover-3xFlag-RbΔCDK protein in early G1 and S/G2 phases under Bortezomib treatment. Related to Fig. S13F.

Figure 5C, three panels:

Anti-UBR5:

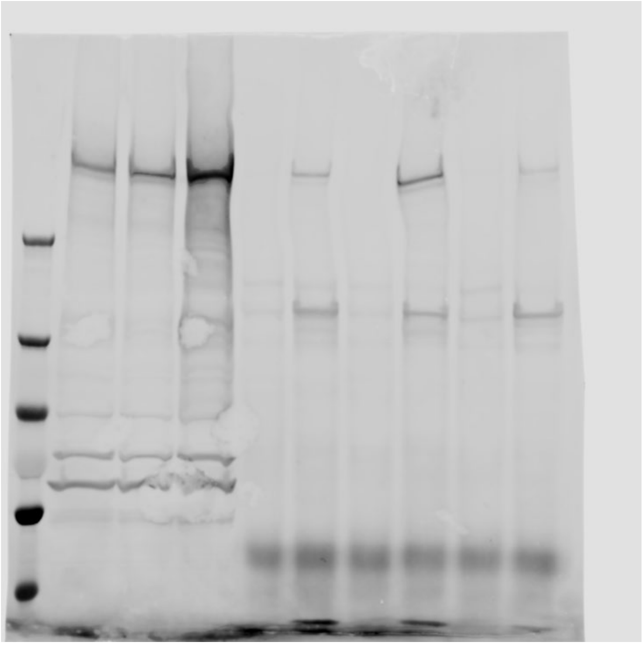

Anti-GFP:

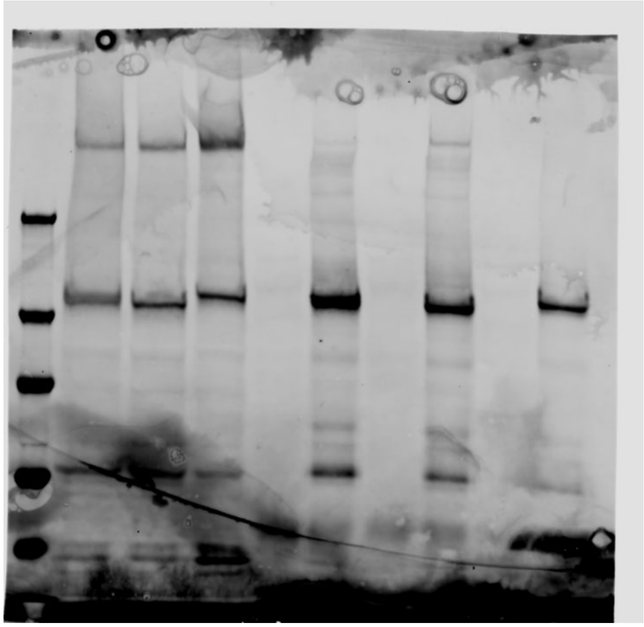

Anti-Flag:

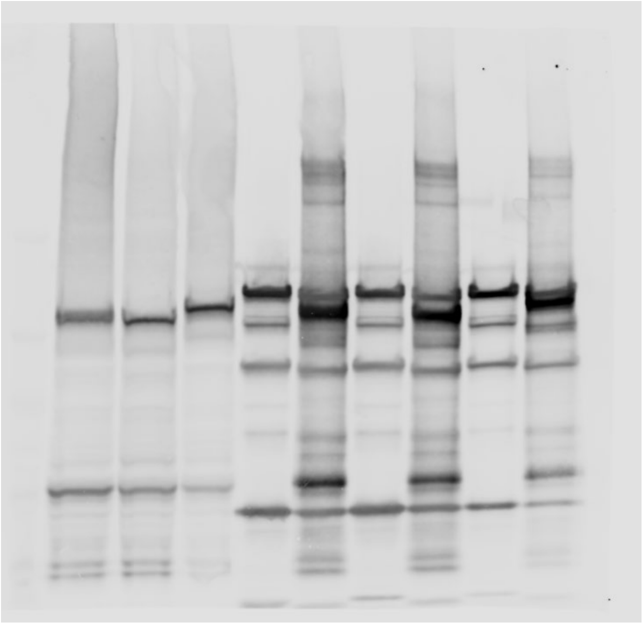

Figure S9C and G, top panels:

Note: I run these two experiments together, so they are on the same gel. To make sure we blot for internal control gene (GAPDH), BTRC knockdown check (anti-BTRC), and Rb on the same gel, we cut the membrane in the middle based on the expected molecular weight.

Anti-Rb (top) and anti-BTRC (bottom):

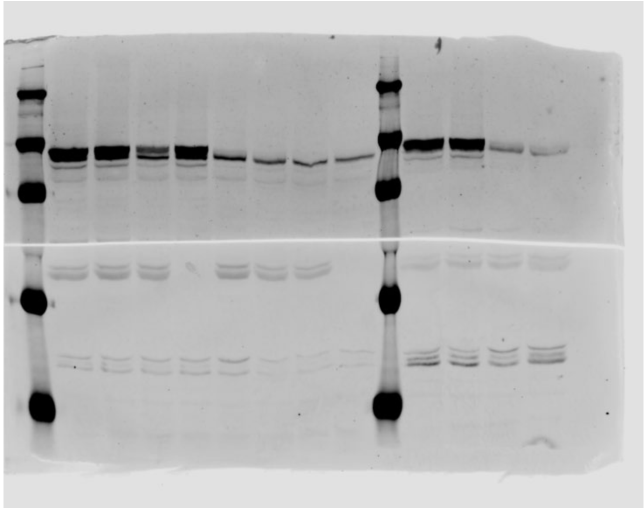

Anti-GAPDH:

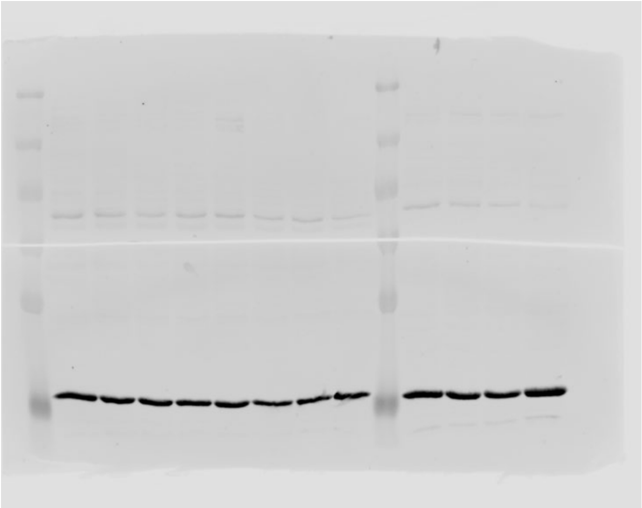

Figure S11E, top panel:
Note: On the right, the two lanes are for another experiment that I was running on the same gel. The left 8 lanes are for the described experiment in Fig. S11E.
To make sure we blot for internal control gene and UBR5 on the same gel, we cut the membrane in the middle based on the expected molecular weight.

Anti-UBR5:

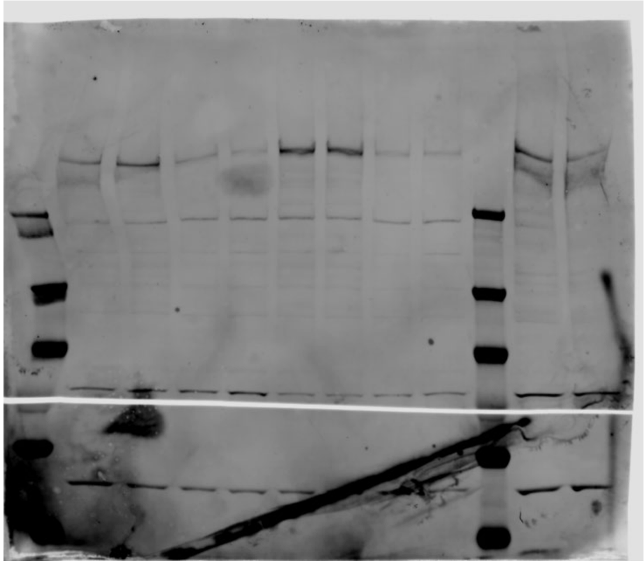

Figure S11G, lower left panel:
Note: The left 8 lanes are for another experiment that I was running on the same gel. The right two lanes are for the described experiment in Fig. S11G.
To make sure we blot for internal control gene (Tubulin) and UBR5 on the same gel, we cut the membrane in the middle based on the expected molecular weight.

Anti-Tubulin:

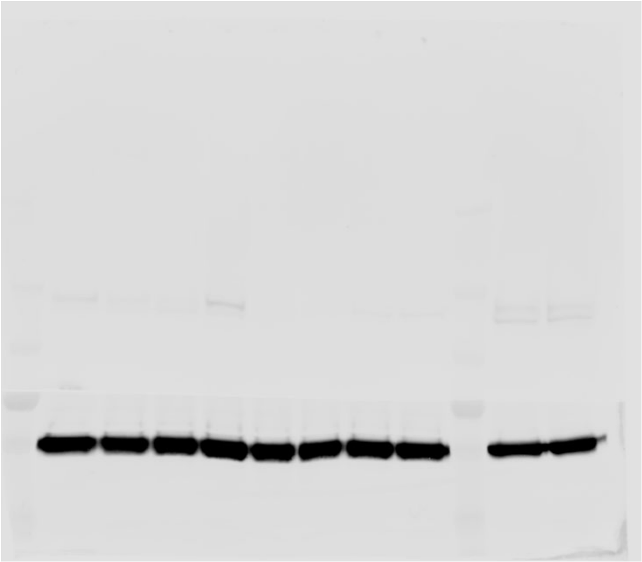

Figure S16A, middle panel:

Note: The left 2 lanes are two controls for Rb expression.
To make sure we blot for internal control gene (Tubulin) and Rb on the same gel, we cut the membrane in the middle based on the expected molecular weight.

Anti-Rb (top) and anti-tubulin (bottom):

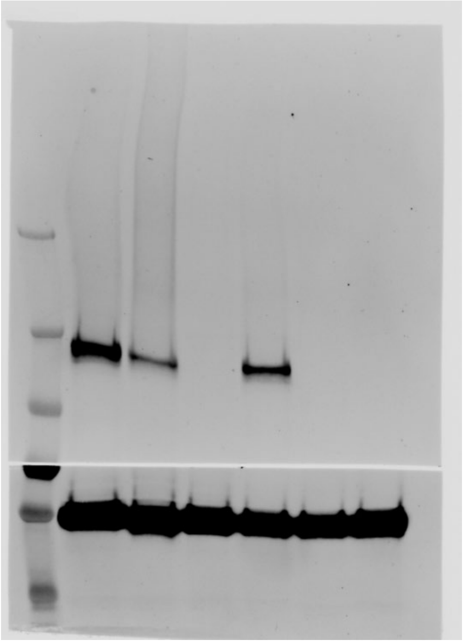

Figure S17A:

Note: The right 2 lanes are 2 samples for another experiment. Also, the full image here is showing all 6 clones we made, and in the manuscript, we showed 3 of them that we picked for the downstream experiment.
To make sure we blot for internal control gene (Tubulin) and UBR5 on the same gel, we cut the membrane in the middle based on the expected molecular weight.

Anti-UBR5:

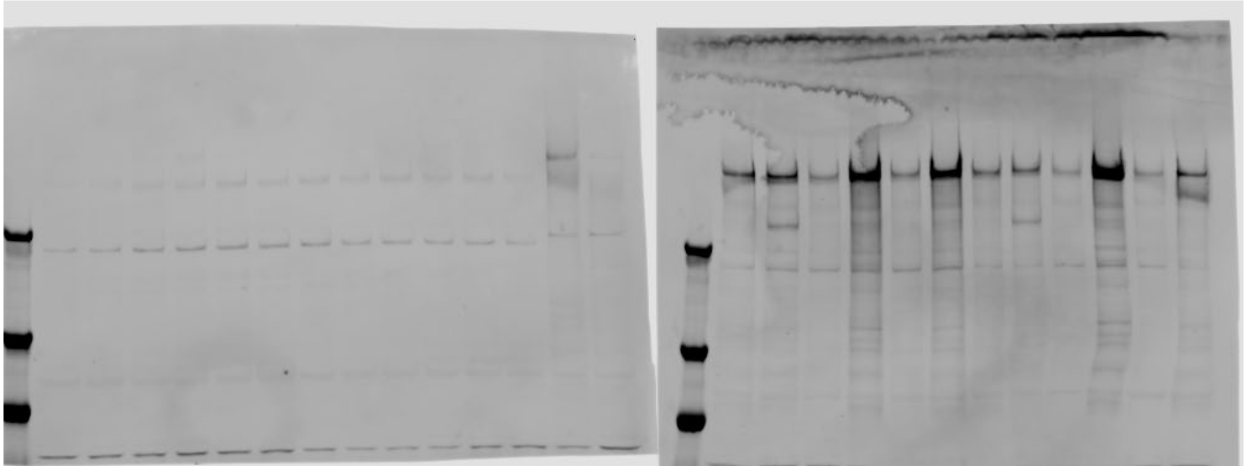

Anti-Tubulin:

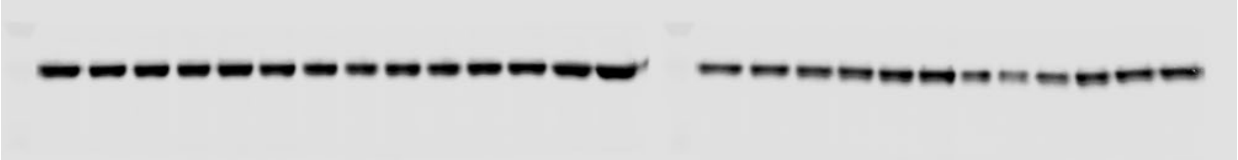

Supplement: Supplementary file 1 — Figs. S1 to S18 Legends for tables S1 to S36 Uncropped Western blots [file sciadv.adq6858_sm.pdf]
